# A Molecular Analysis of Memory B Cell and Antibody Responses Against *Plasmodium falciparum* Merozoite Surface Protein 1 in Children and Adults From Uganda

**DOI:** 10.3389/fimmu.2022.809264

**Published:** 2022-06-02

**Authors:** S. Jake Gonzales, Kathleen N. Clarke, Gayani Batugedara, Rolando Garza, Ashley E. Braddom, Raphael A. Reyes, Isaac Ssewanyana, Kendra C. Garrison, Gregory C. Ippolito, Bryan Greenhouse, Sebastiaan Bol, Evelien M. Bunnik

**Affiliations:** ^1^ Department of Microbiology, Immunology and Molecular Genetics, Long School of Medicine, The University of Texas Health Science Center at San Antonio, San Antonio, TX, United States; ^2^ Infectious Disease Research Collaboration, Kampala, Uganda; ^3^ Department of Infection Biology, London School of Hygiene and Tropical Medicine, London, United Kingdom; ^4^ Department of Chemical Engineering, University of Texas at Austin, Austin, TX, United States; ^5^ Department of Molecular Biosciences and Department of Oncology, Dell Medical School, University of Texas at Austin, Austin, TX, United States; ^6^ Department of Medicine, University of California San Francisco, San Francisco, CA, United States

**Keywords:** malaria, adaptive immune response, humoral immunity, antibodies, memory B cells, IgM, IgG, somatic hypermutation

## Abstract

Memory B cells (MBCs) and plasma antibodies against *Plasmodium falciparum* (*Pf*) merozoite antigens are important components of the protective immune response against malaria. To gain understanding of how responses against *Pf* develop in these two arms of the humoral immune system, we evaluated MBC and antibody responses against the most abundant merozoite antigen, full-length *Pf* merozoite surface protein 1 (PfMSP1_FL_), in individuals from a region in Uganda with high *Pf* transmission. Our results showed that PfMSP1_FL_-specific B cells in adults with immunological protection against malaria were predominantly IgG^+^ classical MBCs, while children with incomplete protection mainly harbored IgM^+^ PfMSP1_FL_-specific classical MBCs. In contrast, anti-PfMSP1_FL_ plasma IgM reactivity was minimal in both children and adults. Instead, both groups showed high plasma IgG reactivity against PfMSP1_FL_, with broadening of the response against non-3D7 strains in adults. The B cell receptors encoded by PfMSP1_FL_-specific IgG^+^ MBCs carried high levels of amino acid substitutions and recognized relatively conserved epitopes on the highly variable PfMSP1 protein. Proteomics analysis of PfMSP1_19_-specific IgG in plasma of an adult revealed a limited repertoire of anti-MSP1 antibodies, most of which were IgG_1_ or IgG_3_. Similar to B cell receptors of PfMSP1_FL_-specific MBCs, anti-PfMSP1_19_ IgGs had high levels of amino acid substitutions and their sequences were predominantly found in classical MBCs, not atypical MBCs. Collectively, these results showed evolution of the PfMSP1-specific humoral immune response with cumulative *Pf* exposure, with a shift from IgM^+^ to IgG^+^ B cell memory, diversification of B cells from germline, and stronger recognition of PfMSP1 variants by the plasma IgG repertoire.

## Introduction

Malaria, caused by the parasite *Plasmodium falciparum* (*Pf*), is responsible for more than half a million deaths every year, of which two-thirds occur in children under the age of five (1). A much larger number of individuals experience non-fatal malaria, amounting to an estimated 241 million cases of disease in 2020. Although the mortality rate for malaria has slowly but consistently declined over the past two decades, the decrease in malaria incidence has plateaued in the past five years. Current interventions are thus insufficient for malaria elimination and novel tools, such as a highly efficacious malaria vaccine, are urgently needed in the fight against this devastating disease. RTS,S, the only malaria vaccine that has elicited protection against (severe) malaria in a phase III clinical trial, had an efficacy against clinical malaria of 30 – 40% in infants and young children (2). Vaccine efficacy was high shortly after vaccination but declined rapidly, and was lower against parasites that were genetically different from strain 3D7 that the subunit vaccine RTS,S was based on (3). Compared to vaccination, repeated natural *Pf* infections eventually elicit superior immunity, consisting of relatively long-lived antibody responses (~2 – 4 years) with cross-strain reactivity (4, 5). This naturally acquired humoral immunity against malaria is associated with the presence of circulating immunoglobulin G (IgG) against *Plasmodium* blood stage antigens (6–13). Recently, immunoglobulin M (IgM) has received increased attention as IgM responses were also shown to correlate with protection and were able to inhibit parasite growth *in vitro* (14–16). A complete understanding of the development of humoral immune responses against *Pf* blood stage antigens over time will help with the development of a more effective and longer lasting vaccine than RTS,S.

Effective and long-lasting humoral immune responses against pathogens consist of long-lived memory B cells (MBCs) in the circulation and plasma cells that reside in the bone marrow and secrete antibody into the circulation [reviewed in (17)]. In the case of *Pf*, long-lived humoral immunity develops over the course of years of repetitive infections, leaving young children susceptible to disease. With cumulative *Pf* exposure, the quality of B cell responses gradually improves. Specifically, the abundance of parasite-specific B cells increases over time (18). In addition, the longevity of plasma cells generated in response to *Pf* infection improves with subsequent exposures (4, 19). Moreover, both MBC and antibody responses gradually broaden to target a larger number of parasite antigens in adolescents and adults (19, 20). Finally, recurrent infections drive the generation of antibodies capable of mediating cross-strain immunity, which is strongly associated with protection (5). Most studies of the development of antibody responses against *Pf* have been performed using serum or plasma, thus precluding the investigation of molecular characteristics of *Pf* -specific antibodies. In addition, little is known about the connection between the MBC and plasma cell compartments. This information would be useful for understanding how durable immunity against malaria develops and may enable us to harness lessons from naturally acquired immunity for improved vaccine design.

**Figure 1:**
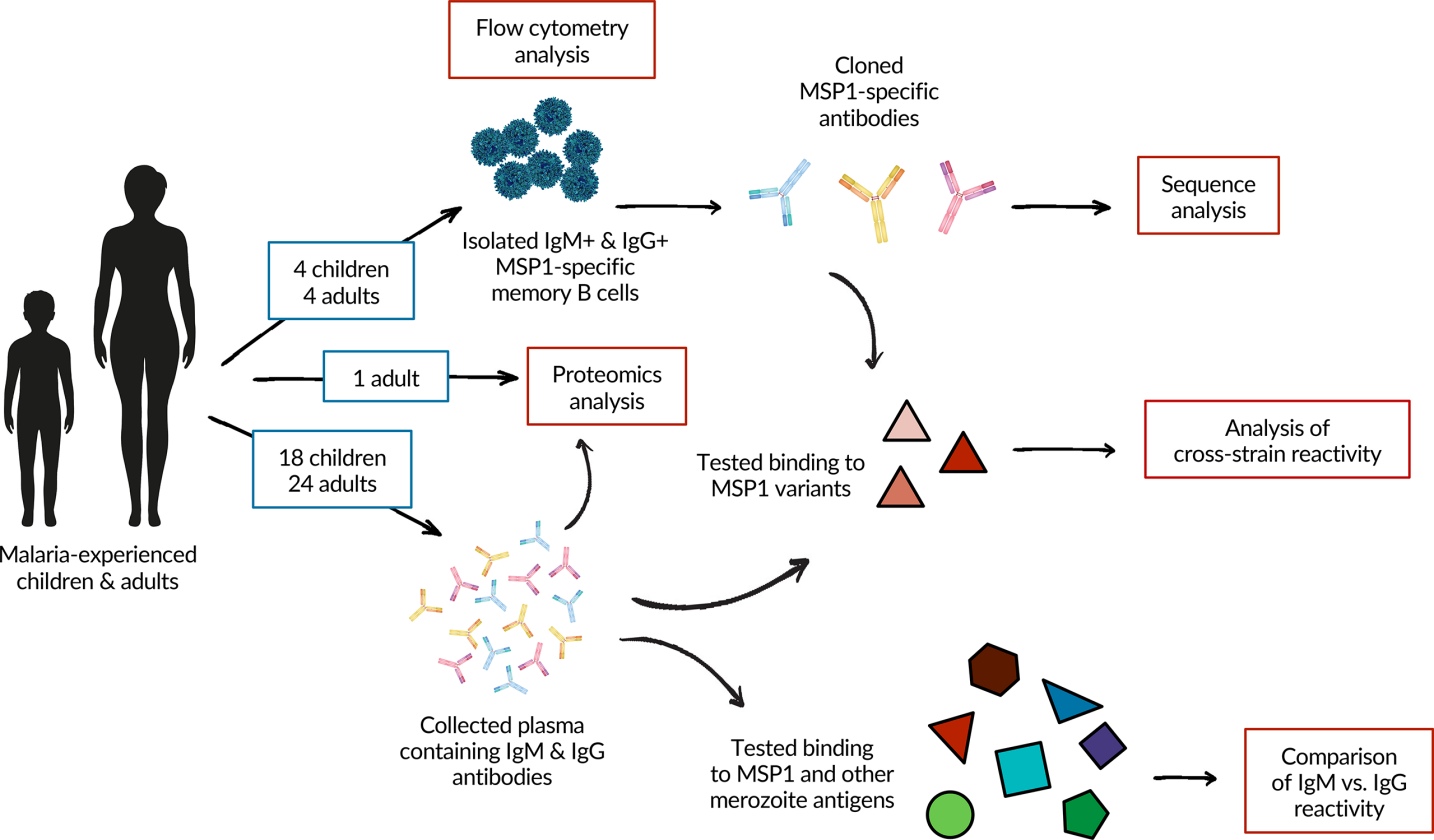
A schematic overview of the experimental approach and analyses performed in this study. A total of 26 adults and 20 children were included in the study. Samples from four children and four adults were used for flow cytometric analysis and the isolation of PfMSP1-specific B cells. Two individuals from each group were also included in the analysis of plasma IgM and IgG responses against PfMSP1 and other merozoite antigens. See **Supplementary Table 1** for details on which donors were included in which experiments.

In this study, we set out to investigate differences in humoral immune responses between children with incomplete protection against malaria and adults who have developed strong immunological protection, to better understand how MBC and antibody responses develop over the course of life-long *Pf* exposure. To do this, we compared the antibody and MBC response against *Pf* antigen merozoite surface protein 1 (PfMSP1) between children and adults living in a region of high *Pf* transmission in Uganda. PfMSP1 is a large, polymorphic, and highly immunogenic protein expressed ubiquitously on the surface of the parasite during the late schizont and merozoite stages (21, 22). PfMSP1 has long been considered a vaccine target since antibody responses against this protein have been associated with protection (6, 21, 23), although results from PfMSP1-based vaccine trials have been disappointing (24, 25). However, since PfMSP1 has high antigenic heterogeneity across *Pf* strains (26, 27), it is a model antigen well-suited to assess antibody cross-strain reactivity elicited by natural infection. In addition, PfMSP1 is commonly used to study *Plasmodium*-specific MBCs in both humans (15, 16, 28, 29) and mice (30–32), and this study therefore adds to a growing body of literature on PfMSP1-specific B cell responses. We studied the isotype of PfMSP1-specific MBCs, characteristics of monoclonal B cell receptor sequences isolated from IgM^+^ and IgG^+^ MBCs, cross-strain reactivity against PfMSP1 variants in both plasma antibodies and B cells, and plasma IgM and IgG reactivity against PfMSP1 and other merozoite antigens (**Figure 1**). In addition, we analyzed the anti-PfMSP1 plasma IgG repertoire and investigated characteristics of antibodies that were found in both the MBC and plasma IgG compartment in more detail.

## Results

### Study Population

To study differences in naturally acquired B cell responses against PfMSP1 between partially immune and immune individuals, we selected children (on average 6.3 years of age, range 4 – 8) and adults (on average 42 years of age, range 22 – 72) residing in Tororo, Uganda, a region with high malaria transmission intensity year-round (**Table 1** and **Supplementary Table 1**) (33). These age ranges were chosen based on pre-existing data about development of immunological protection to malaria for people living in this region (see also Materials and Methods). These individuals were participants of cohort studies, with the exception of two Ugandan adults who were anonymous blood donors. The adult cohort participants had developed immunity against disease, defined as the absence of a malaria episode in the year preceding or following sample collection. All children had at least one febrile malaria episode in the year preceding or following sample collection, but in that time frame also had at least one additional *Pf* infection (either microscopic or submicroscopic) detected during passive surveillance that was cleared in the absence of antimalarial treatment, collectively defined as partial immunity (**Table 1** and **Supplementary Table 1**). All adults and most children were blood smear negative at the time of sample collection, while a considerable proportion of individuals had subpatent levels of parasitemia, with no difference in prevalence between adults and children (**Table 1**). We did not have access to medical information for the two anonymous adult donors, and we included them in the study based on their high levels of plasma antibodies against PfGLURP-R2, PfAMA1, and the C-terminal 19 kDa fragment of PfMSP1 (PfMSP1_19_), suggesting frequent exposure to *Pf* at levels equal to that of the adult cohort participants (**Supplementary Figure 1**).

**Table 1:** Demographic and clinical characteristics of study participants.

	Adult	Children	P value^b^
**Number of Subjects**	26^a^	20^a^	
**Age** (yrs; Median, range)	41 (22 – 72)	6.3 (4.8 – 8.2)	<0.0001
**Sex** (n, %)			0.009
Male	3 (13%)	10 (50%)	
Female	21 (87%)	10 (50%)	
**G6PD genotype** (n, %)			0.77
Wild type	13 (57%)	12 (67%)	
Homozygote	1 (4%)	0 (0%)	
Heterozygote	7 (30%)	5 (28%)	
Hemizygote	2 (9%)	1 (6%)	
**Alpha-thalassemia genotype** (n, %)			0.23
Wild type	11 (48%)	8 (44%)	
Alpha +	9 (39%)	10 (56%)	
Alpha 0	3 (13%)	0 (0%)	
**HbS genotype** (n, %)			0.20
Wild type	18 (77%)	17 (94%)	
Homozygote	0 (0%)	0 (0%)	
Heterozygote	5 (23%)	1 (6%)	
**CD36 genotype** (n, %)			0.43
Wild type	18 (74%)	16 (89%)	
Homozygote	0 (0%)	0 (0%)	
Heterozygote	6 (26%)	2 (11%)	
**Malaria diagnosis at sampling** (n, % positive)	0 (0%)	0 (0%)	>0.99
***Plasmodium* asexual stages by microscopy at sampling** (n, %)	0 (0%)	5 (25%)	0.01
**Submicroscopic *Plasmodium*, by LAMP at sampling** (n, %)	12 (50%)	3 (38%)	0.70
**Malaria diagnosis within one year of sampling date** (n, %)	0 (0%)	20 (100%)	<0.0001
**Parasitemia cleared without treatment within one year of sampling date** (n, %)	23 (96%)	20 (100%)	>0.99

a Sample size within demographic and disease parameters may be less than overall sample size due to missing data, see **Supplementary Table 1**.

b All P values were obtained using a Fisher’s exact test, except for age. Differences in age between the two groups were tested using an unpaired Student’s t-test.

Yrs, years; G6PD, Glucose-6-phosphate dehydrogenase; LAMP, loop-mediated isothermal amplification method.

### PfMSP1-Specific B Cells Have a Classical Phenotype and Are Enriched for IgG in Adults

**Figure 2:**
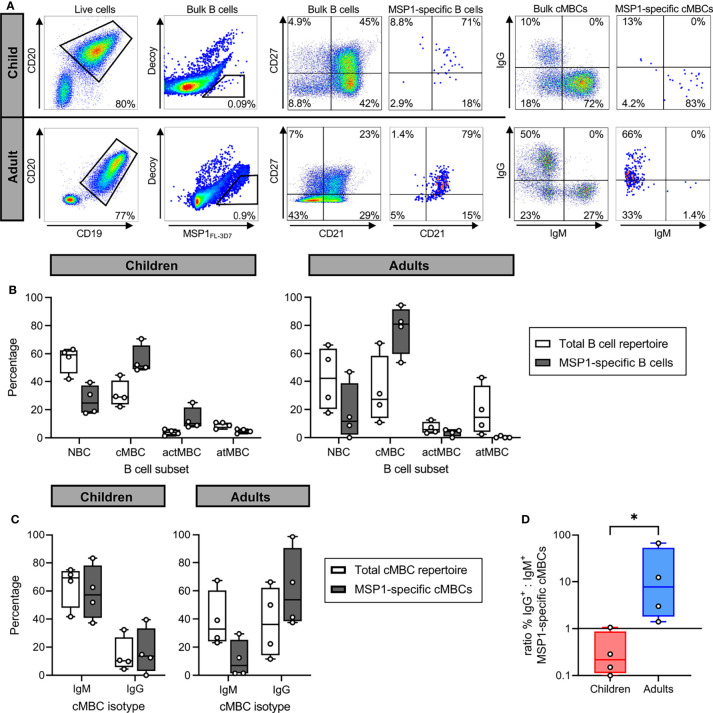
The isotype of PfMSP1_FL-3D7_-specific B cells in malaria-experienced children and adults. **(A)** Representative flow cytometry gating for the sorting and analysis of bulk B cells and PfMSP1_FL-3D7_-specific B cells. B cells were first gated as live CD19^+^CD20^+^ cells, followed by sorting of $PfMSP1_{FL-3D7}^{+}decoy^{-}$ cells. The four major B cell subsets are defined as follows: naïve B cells (NBC), CD21^+^ CD27^-^; classical memory B cells (cMBC), CD21^+^ CD27^+^; activated memory B cells (actMBC), CD21^-^ CD27^+^; and atypical memory B cells (atMBC), CD21^-^ CD27^-^. Data are shown as pseudocolor plots, in which overlapping cells in the plots showing PfMSP1_FL-3D7_-specific B cells and cMBCs are shown in orange and red. **(B)** Relative abundance of major B cell subsets among the total B cell repertoire and among PfMSP1_FL-3D7_-specific B cells in partially immune children (n = 4) and immune adults (n = 4). **(C)** The percentage of IgM^+^ and IgG^+^ B cells among the total repertoire of cMBCs and among PfMSP1_FL-3D7_-specific cMBCs in malaria-experienced children (n = 4) and adults (n = 4). In panels B and C, differences were not tested for statistical significance because the appropriate non-parametric test for paired data (Wilcoxon signed-rank test) does not return a P value lower than 0.13 when using groups of 4. **(D)** The ratio of the percentage of IgG^+^ over IgM^+^ PfMSP1_FL-3D7_-specific cMBCs in malaria-experienced children and adults. A data point with value 0 was plotted at 0.1 for visualization purposes. The difference between groups was tested for statistical significance using a Mann Whitney test. *P < 0.05.

For four children and four adults, cryopreserved PBMCs were used to first isolate bulk B cells, followed by staining with fluorescently labeled tetramers of full-length PfMSP1 from *Pf* strain 3D7 (PfMSP1_FL-3D7_) and decoy tetramers of the irrelevant protein rat CD200 for analysis by flow cytometry, a strategy developed and used by others (30, 34) (**Figure 2A**, second panel). In this approach, PfMSP1_FL-3D7_ tetramers are conjugated to a single fluorochrome (PE), while the decoy tetramers are dual-labeled with PE and AF647 to allow discrimination between PfMSP1_FL-3D7_-specific B cells (PE^+^AF647^-^) and B cells that bind other parts of the tetramer (PE^+^AF647^+^). In both children and adults, PfMSP1_FL-3D7_-specific B cells (defined as $CD19^{+}CD20^{+}\;PfMSP1_{FL-3D7}^{+}decoy^{-}$ ) were predominantly found among CD21^+^CD27^+^ classical memory B cells (MBCs) (**Figure 2B**). This was also observed when analyzing IgM^+^ and IgG^+^ B cells separately (**Supplementary Figure 2**). In addition, in both groups, very few PfMSP1_FL-3D7_-specific B cells were found among CD21^-^CD27^-^ atypical MBCs (**Figure 2B**). To further define the phenotype of PfMSP1_FL-3D7_-specific B cells, we determined the isotype usage of PfMSP1_FL-3D7_-specific classical MBCs. Children showed much larger percentages of IgM^+^ (median, ~70%) than IgG^+^ (median, ~10%) classical MBCs in the total repertoire, which was reflected in the percentage of IgM^+^ and IgG^+^ PfMSP1-specific classical MBCs (**Figure 2C**). In contrast, IgM^+^ and IgG^+^ classical MBCs were equally abundant in adults in the total repertoire, while IgM^+^ PfMSP1-specific classical MBCs were depleted and IgG^+^ PfMSP1_FL-3D7_-specific classical MBCs were slightly enriched (**Figure 2C**). The ratio of IgG^+^ to IgM^+^ PfMSP1_FL-3D7_-specific classical MBCs was higher in all adults as compared to the children (**Figure 2D**). Collectively, these results suggest that the B cell response against PfMSP1 is dominated by IgM^+^ classical MBCs in children, while it is dominated by IgG^+^ classical MBCs in adults. These results are in line with a recent report that showed an increased frequency of IgM usage among *Pf*-specific B cells in young children as compared to older children and adults in Mali, where malaria transmission is highly seasonal (16), suggesting that this is a general feature of the B cell response to *Pf* in children in malaria-endemic regions.

### Recombinant Antibodies Isolated From Memory B Cells Are PfMSP1-Specific and Inhibit Parasite Growth

**Figure 3:**
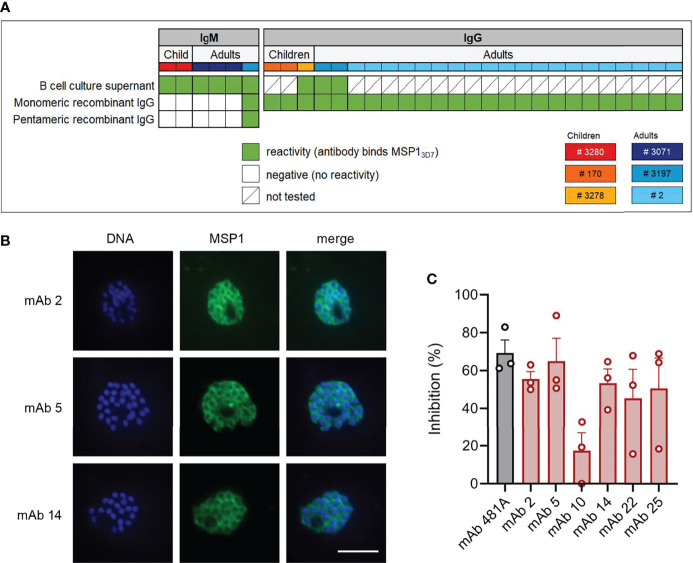
PfMSP1_FL-3D7_ reactivity and growth inhibitory activity of anti-PfMSP1 monoclonal antibodies isolated from memory B cells. **(A)** PfMSP1_FL-3D7_ reactivity of supernatants of clonal MBC cultures determined by ELISA or Luminex assay and reactivity of mAbs obtained from MBCs (recombinant IgG) as determined by Luminex assay. The donor from whom mAbs were isolated is color-coded in the top row. Recombinant mAbs that were derived from IgM^+^ MBCs were tested as both monomeric and pentameric recombinant IgG. **(B)** Immunofluorescence imaging of segmented schizonts using three select mAbs isolated from MBCs of adult donor 2. At this stage, parasites consist of 16 – 32 merozoites, each with its own nucleus (shown in blue), and express PfMSP1 on the parasite cell surface (shown in green). As expected, the overlay shows that PfMSP1 does not colocalize with the nucleus, but instead forms a circular pattern around each individual merozoite. Representative images of two experiments are shown. Scale bar is 5 μm. **(C)** *P. falciparum* growth inhibition by six select mAbs isolated from adult donor 2. Inhibition was calculated relative to a negative control (antibody elution buffer that was buffer exchanged to PBS alongside purified antibodies). Results shown are the average + SEM from three independent experiments. mAb 481A, an anti-PfAMA1 mAb with confirmed growth-inhibitory activity, served as a positive control.

To confirm antigen-specificity of PfMSP1_FL-3D7_-specific B cells, IgM^+^ and IgG^+^ PfMSP1_FL-3D7_-specific B cells were single-cell-sorted into 96-well culture plates containing CD40L-expressing feeder cells, IL-2, IL-21, the TLR-9 agonist ODN2006, and transferrin, which collectively promote B cell survival, expansion, and differentiation into antibody-secreting cells (35, 36). This allowed us to screen B cell clones for antigen-specificity prior to cloning and expression of recombinant mAbs. B cell supernatants were tested for the presence of anti-PfMSP1_FL-3D7_ antibodies by a *Pf* strain 3D7 merozoite ELISA or Luminex assay using recombinant PfMSP1_FL-3D7_. These assays were implemented into our analysis pipeline only in later experiments and these data are therefore missing for samples from donors 170 and 2 as these were analyzed earlier. The variable regions of antibodies with confirmed reactivity to merozoites or recombinant PfMSP1_FL-3D7_ were cloned into linear expression cassettes, expressed as recombinant IgG_1_, and again tested for PfMSP1_FL-3D7_ reactivity by Luminex (**Figure 3A**). In total, we isolated 31 mAbs with reactivity to PfMSP1_FL-3D7_ derived from three children and three adults.

As was reported in a recently published article (15), most anti-PfMSP1_FL-3D7_ IgM antibodies lost their reactivity to PfMSP1_FL-3D7_ when expressed as IgG_1_, which is likely a result of the lower avidity of monomeric IgG as compared to pentameric IgM. We therefore cloned a multimerization domain into the C-terminus of the IgG_1_ heavy chain to express IgM-derived antibodies as pentameric IgG. In contrast to results reported by Thouvenel et al. (15), we were unable to rescue the PfMSP1_FL-3D7_-reactivity of IgM-derived variable regions by expression as pentameric IgG, possibly because we tested only a small number of IgM-derived antibodies (**Figure 3A**). The only pentameric IgG with reactivity to PfMSP1_FL-3D7_ in the Luminex assay was also reactive when expressed in monomeric form. To further confirm the specificity and functionality of the isolated anti-PfMSP1 mAbs, we performed immunofluorescence assays on segmented schizonts for three randomly selected IgG mAbs, showing the expected surface staining of merozoites (**Figure 3B**). We also performed a growth-inhibition assay using blood stage *Pf* (as decribed in (37) with modifications, see Material & Methods) for six IgG mAbs that were selected based on high expression levels in culture. As a negative control, we included antibody elution buffer (100 mM glycine-HCl, pH 2.7) that was buffer exchanged to PBS alongside purified antibodies. mAbs were tested at a single concentration of 200 µg/ml, which is on the low end of what is commonly used to test growth-inhibitory activity of mAbs. The six mAbs showed on average 51% (range, 17% – 65%) inhibition of *Pf* growth as compared to the negative control, demonstrating their ability to inhibit parasite replication (**Figure 3C**).

### PfMSP1-Specific IgG^+^ MBC Lineages From a Malaria-Experienced Adult Are Highly Diversified and Expanded

**Figure 4:**
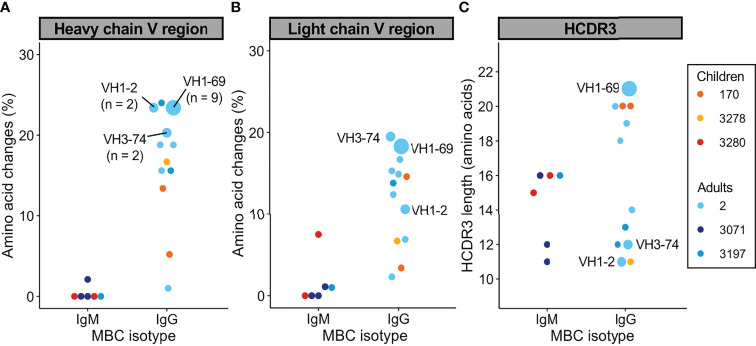
Sequence analysis of the variable heavy and light chains of PfMSP1_FL-3D7_-specific memory B cells. **(A, B)** Percentage of amino acid changes in the V gene segments of the heavy chain **(A)** and light chain **(B)** variable regions. **(C)** Length of HCDR3s. In all graphs, sequences from expanded clonal lineages were grouped. The VH gene usage and size of expanded clonal lineages are indicated.

Next, we set out to study the molecular characteristics of antibodies encoded by PfMSP1_FL-3D7_-specific MBCs. In total, we isolated 6 IgM and 25 IgG mAbs with confirmed PfMSP1_FL-3D7_ reactivity from 6 donors. Full-length heavy and light chain variable regions were obtained and analyzed using IMGT/V-QUEST (38) to determine the level of amino acid substitutions in the V gene segments of the variable regions. PfMSP1_FL-3D7_-specific IgM^+^ MBCs from adults and children carried few amino acid substitutions in both the heavy and light chain V gene segments (average, <1% amino acid changes), while many of the IgG^+^ MBCs were highly mutated (>15% amino acid changes; **Figures 4A, B**). None of the IgM^+^ MBCs were clonally related to each other, defined as sequences using the same heavy chain V and J gene and having a highly similar heavy chain complementarity determining region 3 (HCDR3; ≥85% amino acid similarity), which is likely a result of the very small number of IgM sequences obtained. In contrast, several clonally expanded IgG^+^ MBC lineages were identified in adult donor 2, from whom most mAbs were derived (**Figures 4A, B**). B cells belonging to expanded lineages were among those that were the most diversified from the germline antibody sequences, particularly in the heavy chain V gene segment. Finally, several IgG sequences harbored long HCDR3s (≥ 20 amino acids), while IgM sequences had average or relatively short HCDR3s (**Figure 4C**).

### Recombinant mAbs Isolated From Memory B Cells Recognize Relatively Conserved Epitopes on PfMSP1

**Figure 5:**
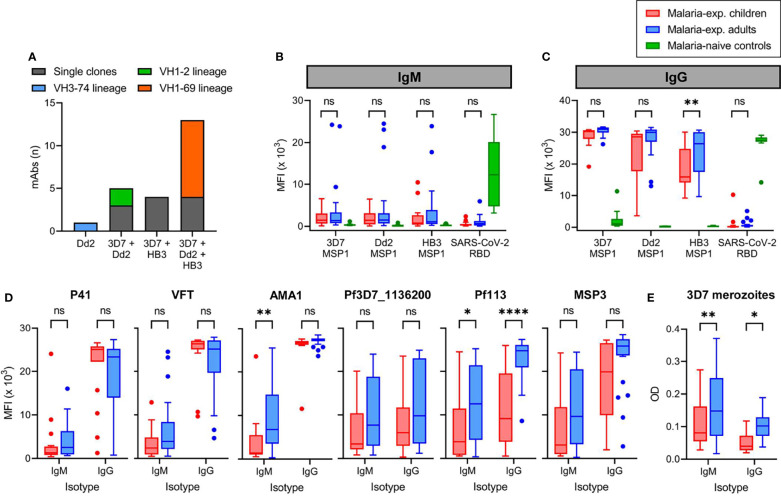
Reactivity of monoclonal antibodies and plasma IgM and IgG against different strains of *P. falciparum.* **(A)** The number of anti-PfMSP1 recombinant monoclonal antibodies (mAbs) with reactivity against recombinant full-length PfMSP1 of three different *P. falciparum* strains in a Luminex assay. Each mAb is color-coded based on whether it was a single clone or derived from an expanded clonal B cell lineages. The VH3-74 lineage consisted of two MBCs, of which we were unable to retrieve the light chain for the second mAb. For this reason, only a single mAb of this lineage was tested here. **(B, C)** Reactivity of plasma IgM **(B)** and IgG **(C)** from malaria-experienced children (n = 18) and adults (n = 24), and malaria-naïve recovered US COVID-19 patients (control; n = 10) against recombinant full-length PfMSP1 of *P. falciparum* strains 3D7, Dd2, and HB3, as well as SARS-CoV-2 receptor binding domain (RBD), as determined by Luminex assay. Values shown are mean fluorescence intensity (MFI). **(D)** Reactivity of plasma IgM and IgG from malaria-experienced children (n = 18) and adults (n = 24) against recombinant *P. falciparum* 3D7 P41, VFT, AMA1, Pf3D7_1136200, Pf113, and MSP3. **(E)** Reactivity of plasma IgM and IgG from malaria-experienced children (n = 18) and adults (n = 24) against whole merozoites by ELISA. Values shown are optical densities after subtraction of background values obtained using pooled unexposed plasma from US donors. Data shown in panels **(B–D)** were tested for statistical significance using a two-way repeated measures ANOVA, followed by comparisons between children and adults using Šídák’s *post-hoc* test, corrected for multiple comparisons. ****P < 0.0001; **P < 0.01; *P < 0.05; ns, not significant.

A study of B cell responses upon influenza virus vaccination reported that the largest clonal B cell families were directed against the most conserved epitopes (39). Based on these observations, we hypothesized that clonally expanded anti-PfMSP1_FL-3D7_ IgG^+^ MBC lineages would have cross-strain reactivity, whereas non-expanded IgG^+^ MBC lineages would be strain-specific. To test this hypothesis, we developed a Luminex assay using beads coated with full-length PfMSP1 variants from the geographically and genetically distinct *Pf* strains 3D7 (African origin; PfMSP1_FL-3D7_), Dd2 (South-east Asian origin; PfMSP1_FL-Dd2_), and HB3 (Central American origin; PfMSP1_FL-HB3_). PfMSP1_FL-3D7_ and PfMSP1_FL-Dd2_ only differ in the sequence of block2, which is the most polymorphic region of PfMSP1, highly immunogenic, and associated with protective antibody responses (40–42), while PfMSP1_FL-HB3_ is divergent throughout most of the protein (**Supplementary Figure 3**). To confirm that measuring reactivity against these PfMSP1 variants in a multiplex format does not lead to unexpected results, we compared the reactivity of several mAbs against these PfMSP1 variants in a multiplex assay versus singleplex assay, yielding a strong correlation between the results (**Supplementary Figure 4**). We then tested 22 IgG^+^ B cell-derived mAbs for binding to the three PfMSP1 variants, while 3 mAbs were excluded due to antibody expression issues. 21 out of 22 mAbs (95%) tested in this assay bound to PfMSP1_FL-3D7_ (**Figure 5A**). This high reactivity with PfMSP1_FL-3D7_ was expected since this protein was used for the isolation of PfMSP1_FL-3D7_-specific B cells. It is therefore unclear why one mAb bound PfMSP1_FL-Dd2_ but not PfMSP1_FL-3D7_. All other mAbs showed reactivity with at least one other PfMSP1 variant, and the majority showed reactivity with both PfMSP1_FL-Dd2_ and PfMSP1_FL-HB3_, including all mAbs from the largest clonal lineage (**Figure 5A**). In contrast to our hypothesis, no obvious difference in breadth of reactivity was observed between the single mAb clones and the expanded mAb lineages. These results suggest that the majority mAbs recognized relatively conserved epitopes, irrespective of the level of clonal expansion of the B cell lineage. Unfortunately, we were unable to determine reactivity of mAbs isolated from IgM^+^ MBCs against the three PfMSP1 variants from different *Pf* strains, because of the loss of antigen reactivity when these mAbs were expressed as IgG. Of note, most mAbs were derived from a single donor, limiting the conclusions we could draw from this experiment.

### Plasma Antibody Responses Against PfMSP1 Are Dominated by IgG in Both Children and Adults

To expand on the results of cross-strain reactivity of mAb against PfMSP1 and to determine whether the PfMSP1_FL-3D7_-specific MBC isotype differences we observed between children and adults are reflected in the plasma antibody response, we tested plasma samples from 24 adults and 18 children for IgM and IgG reactivity against full-length PfMSP1 from the three *Pf* strains (**Supplementary Table 1**). This experimental design provides a measurement of the overall antibody reactivity of the plasma but does not discriminate between antibodies with cross-strain reactivity or a combination of multiple strain-specific antibodies. However, it would reveal whether anti-PfMSP1 antibody responses broaden with cumulative *Pf* exposure. As a control, we included plasma samples from recovered U.S. COVID-19 patients (n = 10) that showed IgM and IgG reactivity against the SARS-CoV-2 receptor binding domain, but not against the three full-length PfMSP1 variants. In both children and adults, anti-PfMSP1 IgM reactivity in the plasma was very low, with the exception of three of the 24 adult samples that contained high IgM reactivity (MFI > 15 × 10^3^) against at least one PfMSP1 variant antigen (**Figure 5B**). In contrast, all samples showed intermediate to high IgG reactivity against all PfMSP1 variants (**Figure 5C**). Adults had higher plasma IgG reactivity against PfMSP1_FL-HB3_ than children. The average reactivity of adult samples against PfMSP1_FL-Dd2_ was also higher but not statistically significantly different from that in children (P = 0.06). These results suggest that while children already have plasma IgG against different variants of PfMSP1, this response continues to broaden with age.

To determine whether the differential IgM and IgG reactivity of plasma is specific for anti-PfMSP1 antibodies or can be extrapolated to antibody responses against other merozoite antigens, we tested reactivity of plasma IgM and IgG to a panel of six recombinant merozoite antigens (all *Pf* 3D7 variants) by Luminex. We observed different reactivity patterns for these antigens (**Figure 5D**). Plasma reactivity to PfP41 and PfVFT was similar to PfMSP1_FL-3D7_, with low IgM and high IgG reactivity in both children and adults. For PfAMA1, IgM reactivity was also low in children, but increased in adults. However, nearly all individuals showed IgM and IgG reactivity against Pf3D7_1136200, Pf113, and PfMSP3, which for Pf113 was higher in adults compared to children (**Figure 5D**). The ratio between IgG and IgM MFI values, where a high ratio is indicative of an IgG-dominant response, was higher for PfMSP1_FL-3D7_ than for each of the six merozoite antigens in adults, and for Pf113, PfMSP3, and Pf3D7_1136200 in children (**Supplementary Figure 5**). Finally, we analyzed serum reactivity to whole merozoites from *Pf* strain 3D7. We observed IgM and IgG reactivity in both groups, with adults showing higher reactivity for both IgM and IgG as compared to children (**Figure 5E**). IgM and IgG anti-merozoite reactivity were not directly comparable since the values measured are dependent on the binding affinity of the secondary antibody. Collectively, these results suggest that in contrast to our observation that the PfMSP1-specific MBC response in children is enriched for IgM^+^ MBCs, the plasma antibody response against PfMSP1 as well as to several other *Pf* merozoite antigens in both children and adults is dominated by IgG. However, based on our experiments with whole merozoites, IgM responses against other merozoite antigens appear better developed, particularly in adults.

### The Anti-PfMSP1 Plasma IgG Repertoire Has Limited Diversity, High Levels of Amino Acid Substitutions, and Mainly Overlaps With Sequences Found in Classical Memory B Cells

**Figure 6:**
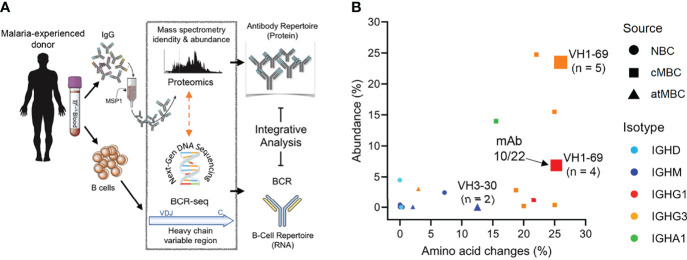
Analysis of the repertoire of anti-PfMSP1_19-3D7_ IgG in the plasma of a malaria-experienced adult. **(A)** Schematic overview of the experimental pipeline. **(B)** The percentage amino acid changes in the V gene segment of the heavy chain variable region (as determined by B cell receptor sequencing; X axis) plotted against the abundance of the corresponding IgG in plasma (Y axis). The size of the data points indicates the number of clonal sequences found in the B cell receptor sequencing (BCR-seq) data set, as a measure of the size of the clonal B cell lineage. The three lineages that were expanded in the BCR-seq data set are indicated with a number, that represents the number of clonal sequences identified by BCR-seq. The shape of the data point shows the B cell subset among which the sequence was found (NBC, naïve B cell; cMBC, classical memory B cell; atMBC, atypical memory B cell), while the color indicates the B cell isotype. The data point corresponding to mAb10 and mAb22 (isolated from PfMSP1-specific B cells) is indicated with an arrow.

To further explore the connection between the plasma cell and MBC compartments of the humoral immune response, we performed an integrative analysis of the plasma anti-PfMSP1 IgG and B cell receptor repertoires in adult donor 2, who was selected based on the availability of additional PBMCs and plasma. Although the results of this experiment will require confirmation in additional individuals, this is the first analysis of its kind in a malaria-experienced person and will provide valuable insight into the molecular characteristics of the anti-PfMSP1 plasma antibody repertoire after life-long exposure to *Pf*. In a previous study, we generated B cell receptor sequencing (BCR-seq) data of antibody heavy chain variable regions of naïve B cells, classical MBCs, and atypical MBCs (43). The full BCR-seq data set and all sequences obtained from PfMSP1-specific MBCs were used to construct a personalized, donor-specific reference heavy chain antibody variable region sequence database. We then isolated anti-PfMSP1 IgG from plasma using commercially available 19 kDa C-terminal fragment of *Pf* strain 3D7 PfMSP1 (PfMSP1_19-3D7_), which limited our analysis to the most conserved domain of PfMSP1 (44, 45). We analyzed the anti-PfMSP1_19-3D7_ IgG preparation by high-resolution liquid chromatography with tandem mass spectrometry and searched the obtained spectra against the donor-specific antibody variable region database (**Figure 6A**). This step allowed us to identify the full-length antibody sequences that these short peptide spectra were derived from. Eighteen anti-PfMSP1_19-3D7_ IgG lineages were identified, of which four lineages made up over 75% of all plasma anti-MSP1_19-3D7_ IgG (**Figure 6B** and **Supplementary Table 2**), suggesting that the anti-PfMSP1_19-3D7_ plasma IgG repertoire may be relatively limited in diversity. The 18 antibody lineages were dominated by sequences that were found among $IgG_{1}^{+}$ and $IgG_{3}^{+}$ classical MBCs, all of which had relatively high levels of amino acid substitutions (>15%) (**Figure 6B**). These results suggest that PfMSP1-specific B cell lineages can give rise to both plasma cells and classical memory B cells, but not atypical MBCs. Two relatively abundant antibody lineages (comprising 23.5% and 6.9% of anti-PfMSP1_19_ plasma IgG) overlapped with IgG^+^ classical MBC lineages that were expanded in the bulk BCR-seq data (5 and 4 clonal B cell members, respectively). Both of these lineages used IGHV1-69 and IGHJ6 and had long HCDR3 sequences of 24 and 21 amino acid residues, respectively, suggesting that these characteristics may have contributed to the selection and expansion of both MBC and plasma cell populations. Long HCDR3s were also observed among the other IgG clonal lineages (average of all lineages, 18 amino acid residues; **Supplementary Table 2**), although the most abundant IgG (representing 24.7% of all anti-PfMSP1_19-3D7_ plasma IgG) had an HCDR3 of 10 amino acid residues. A third antibody lineage with low abundance (<0.1%) matched with an expanded B cell lineage, consisting of two clonal IgM^+^ atypical MBC members that had relatively high levels of amino acid substitutions (>10%). Except for these three expanded lineages, all other clonotypes were found as a single sequence in the bulk BCR-seq data set. These results highlight that the abundance of antibody clonotypes in the plasma is not necessarily reflected by an expansion of the corresponding MBC lineages, pointing towards different selective mechanisms that govern MBC and plasma cell development. It is important to point out that these results may have been influenced by the timing of sampling, and it can be expected to find more overlap between the MBC and plasma antibody repertoires during or shortly after infection.

### Analysis of an Expanded B Cell Lineage Detected in Both Plasma IgG and Memory B Cells

**Figure 7:**
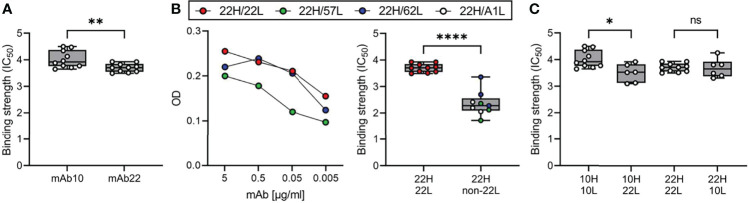
Binding affinity of mAb10 and mAb22 to PfMSP1_3D7_. **(A)** The binding strength of mAb10 and mAb22 as determined by chaotropic ELISA. Binding strength (IC_50_) is defined as the concentration of urea that results in loss of 50% of mAb binding. A total of 10 replicates from three independent experiments are shown. Differences were tested for statistical significance using the Wilcoxon matched-pairs signed rank test, with pairs were defined as antibodies analyzed on the same plate. **(B)** The binding of mAb22 and antibodies that consisted of the heavy chain of mAb22 and a light chain of an unrelated, non-MSP1-reactive antibody to PfMSP1_3D7_ by regular ELISA (left) and chaotropic ELISA (right). Differences in binding strength between replicates of mAb22 (n = 10) and chimeric antibodies (n = 9, three replicates for each of three unrelated light chains that were combined for analysis) were tested for statistical significance using the Mann Whitney test. **(C)** The binding strength of mAb10 and mAb22 (n = 10 replicates each) expressed with their own light chains or chimeric mAbs in which the light chains were swapped (n = 6 replicates each). In all panels, data points are the average of three technical replicates. Differences between antibodies with the same heavy chain but a different light chain were tested for statistical significance using a Kruskal-Wallis test, followed by comparisons between select groups using Dunn’s *post-hoc* test, corrected for multiple comparisons. ****P < 0.0001; **P < 0.01; *P < 0.05; ns, not significant.

To further analyze antibody characteristics that may influence their selection and expansion among plasma cells and MBCs, we compared the anti-PfMSP1_19-3D7_ IgG sequences detected in plasma by mass spectrometry with those derived from the B cell receptors of PfMSP1_FL-3D7_-specific MBCs that were obtained from the same donor at the same time point. Only the largest PfMSP1_FL-3D7_-specific mAb lineage was detected among the anti-PfMSP1_19-3D7_ antibody lineages identified in plasma, potentially because other PfMSP1_FL-3D7_-specific mAbs target epitopes in other parts of the PfMSP1 protein. Alternatively, it is possible that B cell lineages are not often shared between MBCs and plasma cells. Two mAb sequences of this clonal lineage, mAb10 and mAb22, were found in plasma at a percentage of 6.8% and 0.1% of all anti-PfMSP1 IgG detected, respectively, making it the fifth largest clonotype in the circulation. Despite having highly similar heavy chain variable regions, including eight shared amino acid substitutions and a long HCDR3 sequence (21 amino acid residues, **Supplementary Figure 6**), mAb10 and mAb22 had different light chains. Like all other members of the clonal lineage (total n = 9), mAb22 had a lambda light chain, while mAb10 contained a kappa light chain. mAb10 was more abundant in plasma (6.8%), but was the only member of the MBC lineage with a kappa light chain. The opposite was observed for the lambda light chain variants, found at 0.1% of all anti-PfMSP1 plasma IgG, but representing eight out of nine members of the PfMSP1_FL-3D7_-specific MBC lineage. This difference in relative abundance of lineage members in different B cell compartments may be related to the preferential differentiation of high-affinity B cells to plasma cells and lower-affinity B cells to MBCs (46). For mAb10 and mAb22, it would therefore be expected that mAb10 has higher antigen-binding affinity. We determined binding strength of mAb10 and mAb22 to PfMSP1_FL-3D7_ using a chaotropic ELISA with urea and observed that mAb10 indeed showed higher binding strength to PfMSP1_FL-3D7_ than mAb22 (**Figure 7A**). The observation that mAb10 and mAb22 had different light chains suggests that antigen binding by these mAbs is dominated by their heavy chain. To test this, we expressed mAb22 with light chains from unrelated non-PfMSP1_FL-3D7_-binding antibodies and observed that it was still reactive with PfMSP1_FL-3D7_, albeit with lower binding strength (**Figure 7B**). These results suggest that the heavy chain of mAb22 (and presumably mAb10) is sufficient for binding to PfMSP1_FL-3D7_, but that its light chain is important for optimal binding to antigen. This raised the question whether the difference in binding strength between mAb10 and mAb22 is caused by the different light chains used by the two mAbs. For each mAb, we therefore compared binding strength between the antibody expressed with its own light chain and a chimeric antibody in which the light chain was swapped. Expression of mAb10 with the mAb22 light chain resulted in a reduction of binding strength, while the binding strength of mAb22 was unchanged when expressed with the mAb10 light chain (**Figure 7C**). These results suggest that the light chain of mAb10 may play a role in the increased binding strength of mAb10 over mAb22, but is dependent on the mAb10 heavy chain for this effect. Interestingly, despite higher binding strength, mAb10 showed reduced activity in a growth inhibition assay as compared to mAb22 (**Figure 3C**). Collectively, these results highlight characteristics of the individual members of an PfMSP1-specific clonal B cell lineage that may have influenced their selection and fate.

## Discussion

The prevalence and magnitude of antibody responses against *Pf* merozoite antigens have been extensively studied [reviewed in (47, 48)]. In addition, it is known that *Pf* exposure results in the development of long-lived MBCs against merozoite antigens that are maintained in the absence of re-infection (28, 49–51). However, much remains unknown about the molecular characteristics of anti-merozoite antibodies and the connections between the MBC and plasma cell compartments. This information will increase our understanding of how durable B cell immunity develops and how this may be harnessed for vaccine development. Here, we compared the phenotype and molecular characteristics of MBCs against the most abundant merozoite surface antigen, PfMSP1, between a small number of children and adults living in a region of high *Pf* transmission in Uganda. In addition, we analyzed plasma IgM and IgG responses against PfMSP1 and other merozoite antigens in both children and adults from the same region. Finally, we analyzed the overlap between the MBC and plasma antibody compartments using mass spectrometry and B cell receptor sequencing for one adult to better understand the processes that drive B cell selection and fate decisions.

Our results showed that children harbored a larger fraction of PfMSP1-specific IgM^+^ MBCs than adults, in line with a recent report (16). In contrast, the plasma anti-PfMSP1 antibody response was dominated by IgG. We recognize that it is difficult to directly compare IgM and IgG measurements due to potential differences between secondary antibodies used in these assays. However, our control samples from recovered COVID-19 patients demonstrate that we were able to measure IgM reactivity, yet we detected plasma IgM reactivity against PfMSP1 in only a small percentage of adults. These observations suggest that malaria-experienced children develop a strong IgG response against PfMSP1 that is not reflected in the MBC compartment. A similar observation was made by Dorfman et al., who reported a disconnect between IgG responses to PfAMA1 and the presence of PfAMA1-specific IgG^+^ MBCs in children (52). One explanation for the relative lack of IgG^+^ MBCs in children could be that the B cell response predominantly gives rise to short-lived IgG^+^ plasmablasts, whereas germinal center reactions are limited. Since germinal center responses are essential for the development of long-lived plasma cells and MBCs, this would explain the relatively quick waning of anti-parasite plasma IgG and MBCs in children in the absence of exposure, as seen in children living in malaria-endemic regions with a distinct dry season (18, 19). It has been shown that *Pf* infections can lead to dysregulation of various components of the immune system that are essential for the formation of germinal centers, including dendritic cells and T follicular helper cells (53–56). In addition, the abundant development of plasmablasts during *Plasmodium* infection in rodents directly limited the generation of germinal centers as a result of nutrient depletion (57). Thus, IgM^+^ MBCs would mainly develop outside of germinal centers, which would be consistent with the low levels of somatic hypermutation that we observed. An alternative explanation for our observation could be that anti-PfMSP1 IgG^+^ MBCs are formed equally efficiently in both children and adults but undergo recall shortly after their formation in children as a result of repetitive *Pf* infections with high parasitemia, whereas the lower antigenic load of asymptomatic infections in adults would allow for a longer lifespan of IgG^+^ MBCs. This theory would be in line with the increase in the percentage of merozoite-specific MBCs with age that is observed in individuals who live in malaria-endemic regions (18). In support of this theory is our observation that the few IgG^+^ MBCs isolated from children already had relatively high levels of amino acid substitutions, suggestive of multiple rounds of affinity maturation in germinal centers. It is also supported by our observation of a lack of clonal connections between IgG^+^ MBCs at two time points six months apart in two five-year old children living in a high transmission region in Uganda, while we did find clonally related sequences among IgM^+^ MBCs between the same two time points (58). These data could suggest that the IgG^+^ MBC compartment in children undergoes more rapid turnover than the IgM^+^ MBC compartment, or that the IgM^+^ MBC compartment has more self-renewing capacity (59). Of note, our findings may be specific for PfMSP1 and the small sample size in many of our experiments precludes definitive conclusions. More longitudinal studies in the same individuals will be needed to track the fate of parasite-specific IgG^+^ MBCs after their formation.

We observed a low level of plasma IgM against PfMSP1 as compared to plasma IgG. Although IgM reactivity was higher against other *Pf* antigens, the question of how much IgM contributes to the control of *Pf* infections remains unanswered. IgM responses against a variety of *Pf* antigens have been reported in several studies, in particular in high transmission regions (60–63), but there is conflicting evidence on the durability of the IgM response and its role in parasite inhibition. Boyle et al. reported that IgM responses against PfMSP2 were sustained in children living in Kenya during a period with low parasite transmission (14). In contrast, Walker et al. concluded that IgM responses against five other merozoite antigens decreased rapidly following *Pf* malaria in children living in Ghana, whereas IgG responses against these five antigens were maintained for a longer period of time (63). These conflicting observations may be the result of differences between the assays used to assess these antibody responses, or differences in the development of IgM responses under different transmission intensities or against different parasite antigens, as also observed in this study. An association between IgM reactivity against *Pf* antigens, including PfMSP1_19_, PfAMA1, PfMSP3, and PfGLURP, and protection against malaria was reported by some (14, 64, 65), but not by others (66). When tested at the same concentration, purified plasma IgM from malaria-experienced individuals had equal opsonic phagocytosis activity and two-fold lower fixation capacity of C1q, the primary component of the classical complement pathway, as compared to plasma IgG, but induced nine-fold higher deposition of components of the membrane attack complex (14, 16). While these results suggest that IgM can indeed contribute to parasite inhibition, the concentration of total IgM in plasma is almost one order of magnitude lower than that of IgG [average, 1.5 g/l for IgM versus 11 g/l for IgG (67, 68)]. Therefore, the relative contributions of IgM and IgG to parasite inhibition remain to be determined. Finally, a passive immunization study showed that IgG-depleted plasma from malaria-experienced adults had no effect on parasitemia in children with *Pf* malaria, whereas treatment with IgG from the same individuals resulted in a dramatic decrease of parasite counts (69), suggesting that IgG is the main effector antibody responsible for parasite control.

The acquisition of high levels of amino acid substitutions in IgG^+^ MBCs from both children and adults suggests that these cells are the product of multiple rounds of affinity selection. We also detected several B cells with long HCDR3s, similar to broadly neutralizing antibodies against HIV (70), although this was not a universal characteristic of anti-MSP1 mAbs. In addition, we observed that children and adults had equally high IgG reactivity against PfMSP1_FL-3D7_, but that reactivity against PfMSP1_FL-HB3_ was lower in children. This suggests that the IgG response continues to broaden with subsequent *Pf* infections, resulting in a larger fraction of IgG directed against conserved epitopes. The broadening of antibody responses, high mutational load, and expansion of clonal PfMSP1-specific B cell lineages is suggestive of strong selection pressure on the B cell response as a result of *Pf* infections.

We believe that our observations regarding mAb10 and mAb22, two members of an PfMSP1-specific IgG^+^ MBC lineage that share the same heavy chain but have different light chains, are additional evidence of strong forces on B cell selection and development. The difference in light chain usage between mAb10 and mAb22 raises the question how these mAbs are developmentally related. One possibility is that mAb10 and mAb22 were derived from a B cell precursor that underwent cell division upon heavy chain rearrangement in the bone marrow, giving rise to multiple daughter cells that each underwent light chain rearrangement independently. In this scenario, the shared amino acid mutations would be the result of convergent evolution selecting for higher affinity antigen binding. In a second scenario, a precursor B cell activated by antigen could have undergone secondary light chain rearrangement in the germinal center. In this case, the kappa light chain-bearing mAb10 would likely be evolutionarily closer to this precursor, and mAb22 (and all other MBCs in this lineage) would be derived from this secondary rearrangement event and have undergone further diversifying selection. We were unable to determine whether mAb10 and mAb22 developed as a result of convergent evolution or clonal lineage diversification. However, our results suggest that the light chains of these antibodies may affect antibody binding affinity. This could, on its own, have resulted in the preferential differentiation of the higher affinity mAb10 variant into a plasma cell and the lower affinity mAb22 variant into an MBC. Further studies into the relationship between the plasma cell and MBC compartments for PfMSP1 and other merozoite antigens are currently ongoing in our laboratories.

Given its higher binding affinity, the relatively low inhibition of parasite growth by mAb10 as compared to mAb22 is unexpected. One potential explanation is that inhibition as measured in the growth inhibition assay can be the result of multiple mechanisms, such as neutralization or prevention of proteolytic cleavage of PfMSP1. High binding affinity is likely to be important for optimal neutralization. For inhibition of proteolytic cleavage, on the other hand, the precise antibody epitope and orientation may be more important determinants. The kappa and lambda light chains of mAb10 and mAb22 may have different footprints on the surface of PfMSP1, thereby differentially occluding the cleavage site that releases PfMSP1_19_, resulting in differences in growth inhibition. It will be interesting to determine the ability of these antibodies to induce opsonic phagocytosis or fix complement, as these measurements of parasite inhibition may be influenced more strongly by antibody affinity.

This study has several limitations. First, we analyzed PfMSP1-specific B cell phenotypes in a small number of individuals. However, the observed shift from predominantly IgM^+^ PfMSP1-specific MBCs in children to IgG^+^ PfMSP1-specific MBCs in adults was in agreement with results reported recently by Hopp et al., which provides strength to our observations. Second, the sequence analysis of mAbs cloned from PfMSP1-specific B cells and anti-MSP1 plasma IgG were performed using a small number of donors and small numbers of antibody sequences. We used stringent criteria for the inclusion of mAbs in downstream analyses and only analyzed the sequences of mAbs with confirmed PfMSP1 reactivity, which may have affected our observations with regard to antibody diversity and clonal expansion. Finally, we selected PfMSP1 as a model antigen for this study. An analysis of plasma antibody responses against other merozoite antigens highlighted differences in the plasma antibody response between PfMSP1 and PfMSP3, Pf113, and Pf3D7_1136200 (**Figure 5D**), suggesting that the results reported here apply to PfMSP1, but may not necessarily be representative of B cell responses to other *Pf* antigens.

In conclusion, we performed an analysis of the MBC compartment and antibody responses against PfMSP1 in children and adults living under high *Pf* transmission conditions. We observed that children in our study predominantly harbor PfMSP1-specific IgM^+^ MBCs, while the MBC response shifted to IgG^+^ B cells in adults. In contrast to this difference in isotype among PfMSP1-specific MBCs, both children and adults demonstrated strong anti-MSP1 plasma IgG responses, while anti-PfMSP1 plasma IgM responses were minimal. IgG^+^ MBCs carried high levels of somatic hypermutations and clonal expansion, suggestive of ongoing B cell selection over the course of sequential *Pf* infections. Finally, we directly compared the overlap between the PfMSP1-specific MBC compartment and anti-PfMSP1 plasma IgG, revealing that the same molecular characteristics observed in the MBC compartment were also dominant in the plasma IgG compartment. Collectively, our results provide new insights into the development of B cell responses against *Pf*, in particular about the similarities and differences between MBC and antibody responses.

## Materials and Methods

### Ethics Approval Statement

Participants were enrolled in the Tororo Child Cohort or the Program for Resistance, Immunology, Surveillance, and Modeling of Malaria (PRISM) Cohort, and provided written consent for the use of their samples for research. These cohort studies were approved by the Makerere University School of Medicine Research and Ethics Committee (SOMREC) and the University of California, San Francisco Human Research Protection Program & Institutional Review Board. The use of cohort samples was approved by the Institutional Review Board of the University of Texas Health Science Center at San Antonio. Donors 1 and 2 were anonymous blood donors at Mbale regional blood bank in Eastern Uganda, who consented to the use of their blood for research. The use of samples from anonymous blood donors was not considered human research by the Institutional Review Board of the University of Texas Health Science Center at San Antonio due to the lack of any identifiable information and was therefore exempt from review.

COVID-19 samples used in this study were received de-identified from the University of Texas Health San Antonio COVID-19 Repository. This repository was reviewed and approved by the University of Texas Health Science Center at San Antonio Institutional Review Board. All study participants provided written informed consent prior to specimen collection for the repository to include collection of associated clinical information and use of left-over clinical specimens for research. The COVID-19 Repository utilizes an honest broker system to maintain participant confidentiality and release of de-identified data or specimens to recipient investigators.

### Participants

Malaria-experienced participants (n = 46) were residents of Eastern Uganda, a region with extremely high malaria transmission intensity (annual entomological inoculation rate in the region estimated at 125 infectious bites per person per year (71). In this region, children between five and ten years of age start to develop protective immune responses against malaria, as evidenced by a record of parasitemia in the absence of malaria, but are typically still susceptible to disease. Children above ten years of age and adults have developed protective immune responses, evidenced by no signs of clinical malaria despite high exposure, demonstrated by household entomological and epidemiological measures, including documented asymptomatic parasitemia. In addition to selection based on age, sex was used as an inclusion criterium to ensure equal representation of males and females among the children, and to include as many males as possible for the adults. The PRISM cohort provided health facility-based malaria surveillance. All children 6 months to 10 years old within a household and up to one primary adult caregiver (often female) were enrolled in a dynamic cohort, and children were excluded from follow-up after reaching 11 years of age. Participants had routine clinic visits roughly every 3 months and attended a study clinic any time they became ill.

COVID-19 patients (n = 10) were enrolled in the University of Texas Health San Antonio COVID-19 Repository. Blood was collected after discharge from the hospital, on average approximately 30 days after symptom onset. These donors were included as malaria-naïve controls.

### MSP1 Expression and Tetramer Synthesis

To produce C-terminal biotinylated full-length 3D7 PfMSP1 (MSP1_FL-3D7_), human Expi293F cells (Thermo #A14635) were cultured, passaged, and transfected with the plasmids MSP1-bio (Addgene #47709) and secretedBirA-8his (Addgene #32408) at a 4:1 (w/w) ratio according to Thermo’s protocol. Both plasmids were a kind gift from Gavin Wright (72, 73). Biotin (Thermo #PI21336) was added to a final concentration of 100 µM immediately after adding the transfection mix. Cell were cultured in either non-baffled polycarbonate flasks with a vent cap (Fisher #PBV12-5) or in glass Erlenmeyer flasks loosely covered with aluminum foil to allow for gas exchange. Cells were successfully passaged in volumes as low as 5 ml. Absence of mycoplasma contamination was confirmed using the MycoAlerta Plus mycoplasma detection kit (Lonza #LT07705). Culture supernatants were collected 5 – 7 days post-transfection by centrifuging the culture at 4,000 × g for 25 min. at RT. A 10 kDa cutoff Protein Concentrator PES (Thermo #88527) was used (5,000 × g at 4°C) to exchange culture medium containing free biotin for PBS (pH 7.2) (> 100,000 dilution) and to concentrate the protein to a final volume of 0.5 – 1 ml. The 3D7 PfMSP1 protein was mixed with 6 – 12 volumes of PBS (pH 5.5) in a final volume of six ml and was subsequently loaded onto gravity flow columns (Thermo #29924) containing CaptAvidin agarose (Thermo #C21386) for purification. After three washes with PBS (pH 5.5) and five 6 ml elutions with PBS (pH 10.5), the elutions were pooled (30 ml) and the pH was immediately neutralized by adding 12 ml PBS (pH 5.5). After concentrating, the protein was quantified using the Coomassie Plus (Bradford) Assay Kit (Thermo #23236) on a NanoDrop One spectrophotometer, according to the manufacturer’s instructions, visualized by SDS-PAGE (see methods below; **Supplementary Figure 7A**), diluted to 1 mg/ml, aliquoted, and stored at -70°C.

Since each streptavidin molecule has the ability to bind four biotinylated PfMSP1_FL-3D7_ molecules, PfMSP1_FL-3D7_ tetramers were made by incubating PfMSP1_FL-3D7_ in a tube revolver (Thermo #88881001) at 40 rpm and RT for 30 min. with streptavidin-PE (Thermo #S866) at a 6:1 molar ratio. After this incubation, the tetramers were washed with PBS (pH 7.2) using a Vivaspin centrifugal concentrator (Sartorius #VS0141) three times for 5 min. at 15,000 × g at RT. To make decoy tetramers, streptavidin-PE was first conjugated to Alexa-fluor 647 (Thermo #A20186) per manufacturer’s instructions. This double-conjugated streptavidin was then coupled to *R. norvegicus* CD200 [Addgene #36152 (73)] as described above.

### SDS-PAGE

As a quality control step, purified proteins were visualized on a polyacrylamide gel. Purified samples were mixed with Laemmli buffer and NuPage sample reducing agent (Thermo #NP0004; not added for monoclonal antibodies), incubated at 85°C for two min. The samples were run on a 4 – 12% Bis-Tris gel (Thermo #NP0321BOX) with MOPS running buffer (Thermo #NP0001) at 200 V for 50 min. The proteins were stained using Imperial Coomassie protein stain (Thermo #PI24615) per manufacturer’s instructions.

### MSP1-Specific B Cells Isolations

Peripheral blood mononuclear cells (PBMCs) from malaria-experienced individuals were cryopreserved in liquid nitrogen at -196°C and continuously kept at this temperature during storage and transport to minimize changes to B cell phenotypes (74). Cryopreserved PBMCs from malaria-experienced children and adults were thawed in a water bath at 37°C and immediately mixed with pre-warmed thawing medium [IMDM Glutamax (Thermo #31980030) supplemented with 10% heat-inactivated fetal bovine serum (FBS) of US origin (Sigma #TMS-013-B) and 33 U/ml universal nuclease (Thermo #88700)] and then centrifuged (5 min. at 250 × g and RT). The cell pellet was resuspended in thawing medium and viable cells were counted by adding 10 µl filtered 0.2% trypan blue in PBS to 10 µl of the cell suspension on a Cellometer Mini (Nexcelom) automated cell counter. Next, cells were pelleted by centrifugation (5 min. at 250 × g and RT) and resuspended in isolation buffer (PBS supplemented with 2% heat-inactivated FBS and 1 mM EDTA) at 50 million live cells/ml and filtered through a 35 μm sterile filter cap (Corning #352235) to break apart any aggregated cells. B cells were isolated using StemCell’s EasySep Human B Cell Isolation Kit (#17954) according to manufacturer’s instruction. After washing with PBS, the isolated B cells were incubated with 1 µl LIVE/DEAD Fixable Aqua Dead Cell Stain Kit (Thermo #L34965) per 1 ml cell suspension, per manufacturer’s instructions. After washing the B cells with cold PBS and resuspending them in 50 µl cold PBS with 1% bovine serum albumin (BSA) (Sigma #A7979), the cells were first stained with 40 nM of decoy tetramer (10 min. in the dark on ice) and then with 20 nM of PfMSP1_FL-3D7_ tetramer (30 min. in the dark on ice), followed by a wash with 1 ml of cold PBS/1% BSA (5 min. at 250 × g and RT). Tetramer-bound B cells were selected using StemCell’s EasySep Human PE Positive Selection Kit (#17664) and subsequently stained on ice for 30 min. with an antibody panel against B cell surface markers (**Supplementary Table 3**). UltraComp eBeads (Thermo #01222242) were used to prepare compensation controls for each fluorophore per manufacturer’s instructions. Before acquisition on a BD FACSAria II cell sorter, the cells were washed with 3 ml of cold PBS with 1% BSA (5 min. at 250 × g and 4°C), diluted to 20 – 30 million cells/ml in PBS with 1% BSA, and filtered into a FACS tube with filter cap. Lymphocytes were gated using forward and sideward scatter, followed by doublet exclusion and gating on live cells. PfMSP1-specific mature IgG^+^ and IgM^+^ B cells (CD19^+^, CD20^+^) were gated (PE^+^, AF647^-^) and single cells were sorted into 100 µl IMDM/Glutamax/10% FBS in a well of a 96-well plate (Corning #353072). One day prior to the sort, each well was seeded with 30,000 adherent, CD40L-expressing 3T3 cells (kind gift from Dr. Mark Connors, NIH) in 100 µl IMDM/Glutamax/10% FBS containing 2× MycoZap Plus-PR (Lonza #VZA-2021), 100 ng/ml human IL-2 (GoldBio #1110-02-50), 100 ng/ml human IL-21 (GoldBio #1110-21-10), 5 µg/ml TLR9-activator ODN2006 (IDT DNA, sequence TCGTCGTTTTGTCGTTTTGTCGTT), and 60 µg/ml transferrin (Sigma #616424) to promote expansion and differentiation of B cells into antibody-secreting cells (35, 36). After incubation at 37°C and 8% CO_2_ for two weeks, the wells were screened for the production of IgM or IgG by enzyme-linked immunosorbent assay (ELISA).

### Parasite Culture and Merozoite Isolation

*Pf* strain 3D7 parasites were cultured (75) in human AB^+^ erythrocytes (Interstate Blood Bank, Memphis, TN, USA) at 3 – 10% parasitemia in complete culture medium (5% hematocrit). Complete culture medium consisted of RPMI 1640 medium (Gibco #32404014) supplemented with gentamicin (45 µg/ml final concentration; Gibco #15710064), HEPES (40 mM; Fisher #BP3101), NaHCO_3_ (1.9 mg/ml; Sigma #SX03201), NaOH (2.7 mM; Fisher #SS266-1), hypoxanthine (17 µg/ml; Alfa Aesar #A11481-06), L-glutamine (2.1 mM; Corning #25005Cl), D-glucose (2.1 mg/ml; Fisher #D16-1), and 10% heat-inactivated human AB^+^ serum (Valley Biomedical #HP1022). Parasites were cultured at 37°C in an atmosphere of 5% O_2_, 5% CO_2_, and 90% N_2_. Before use in cultures, 12.5 ml packed erythrocytes were washed twice with 10 ml cold incomplete medium (complete culture medium without human serum) and pelleted between each wash by centrifugation at 500 × g for 8 min. at 4°C (max. acceleration and weakest break). Washed erythrocytes were resuspended in 2 volumes of complete medium and stored at 4°C.

Parasites were synchronized to the ring stage by treatment with 5% D-sorbitol (76) (Fisher #S459-500). Cultures containing high percentages of ring-stage parasites were centrifuged at 250 × g for 5 min. at RT. Pelleted erythrocytes were resuspended in 10 volumes of 5% D-sorbitol in MQ water, vortexed for 30 sec. and incubated for 8 min. at 37°C. The cells were then washed with 5 volumes of complete culture medium (250 × *g* for 5 min. at RT) and resuspended in complete culture medium at 5% hematocrit and cultured as described above. To obtain tightly synchronized parasites, sorbitol treatments were performed twice, 14 hours apart.

Infected erythrocytes containing parasites in the late-trophozoite and schizont stages were isolated from culture by magnetic separation (77, 78). Late-stage parasites were separated from uninfected and ring-infected erythrocytes with a SuperMACS II Separator (Miltenyi #130-044-104). The magnet was assembled with a D column (Miltenyi #130-041-201) according to manufacturer’s instructions. The column was equilibrated with 200 ml incomplete medium. An additional 50 ml incomplete medium was added to the column through the side syringe to remove air bubbles possibly remaining in the column matrix. A 22 G needle (BD #305155) was attached to the stopcock to serve as a flow restrictor. For safety purposes the plastic protective sheath remained on the needle after cutting the end to allow flow of the liquid without exposing the tip of the needle. Approximately 100 – 200 ml of synchronized parasite culture (5 – 10% parasitemia, 5% hematocrit) 24 – 27 hours following the second sorbitol treatment (majority of parasites in the early segmented schizont stage, 4 – 6 nuclei visible by Giemsa staining) were used for merozoite isolation. After passing the parasite culture through the column, the column was washed from the top with incomplete medium until the flowthrough was clear (usuall ~100 ml). Next, the column was washed with a total of 150 ml incomplete medium (50 ml from the side and 100 ml from the top). Erythrocytes containing late-stage parasites with high paramagnetic hemozoin levels are preferentially retained in the column matrix while attached to the magnet (79) allowing for separation of late-stage parasites from uninfected erythrocytes and early-stage parasites. The column was removed from the magnet and 60 ml incomplete medium was used to elute the erythrocytes from the column matrix. The erythrocytes were pelleted by centrifugation at 250 × *g* for 5 min. at RT and were resuspended in 3 ml complete culture medium. Infected erythrocytes were incubated with E64 (10 µM final concentration, Sigma #324890-1MG) for 8 hours at normal culture conditions to allow the parasites to develop into fully segmented schizonts while preventing egress from the erythrocytes. Infected erythrocytes containing schizonts were then pelleted by centrifugation at 1,900 × *g* for 8 min. at RT and the supernatant containing E64 was removed. A thin smear from the pellet was Giemsa stained and merozoite yield was assessed by counting the number of fully segmented schizonts present. The pellet was resuspended in 4 ml incomplete medium. Merozoites were released from the erythrocytes by passing them through a 1.2 µm syringe filter (Pall #4190) and were subsequently pelleted by centrifugation at 4,000 × *g* for 10 min. at RT. On average, 5 × 10^7^ merozoites were collected per 25 ml of synchronized culture. The merozoites were resuspended in PBS and stored at 4°C for up to one day until used for the ELISA.

### Enzyme-Linked Immunosorbent Assays

To detect IgG and IgM, 96-well ELISA plates (Corning #3361) were coated with either goat anti-human IgG (Sigma #I2136) or IgM (Sigma #I1636) antibody at a concentration of 4 and 8 µg/ml, respectively, diluted in PBS, at a total volume of 100 µl per well. After a one hour incubation at 37°C or O/N at 4°C, each well was washed once using slowly running (approximately 900 ml/min.) deionized water. This washing method resulted in significantly higher specificity than other methods tested in the lab (using a plate washer with water or PBS containing 0.1% tween-20, or a squeeze bottle filled with PBS containing 0.1% tween-20). All subsequent washes were performed this way. 150 µl blocking buffer (one-third Non-Animal Protein (NAP)-Blocker (G-Biosciences #786-190P) and two-thirds PBS) was added to each well to prevent non-specific binding. After one hour of incubation at 37°C, the wells were washed three times and 50 µl B cell culture supernatant diluted 1:1 in dilution buffer (1% NAP Blocker in PBS; total volume 100 µl) was added per well. Plates were incubated for two hours at 37°C and washed five times. Then, either 100 µl 1:2500 diluted (1% NAP Blocker in PBS) HRP-conjugated anti-human IgG antibody (BioLegend #410902) or 1:5000 HRP-conjugated anti-human IgM antibody (Sigma #AP114P) was added to each well. After incubation for one hour at 37°C and three washes, HRP activity was detected using 50 µl TMB (Thermo #PI34024). Plates were incubated in the dark at RT and the oxidation reaction was stopped by adding 50 µl 0.18M H_2_SO_4_ (Fisher #FLA300-212) per well when the negative controls (wells that received buffer when test wells received culture supernatant) started to color. Absorbance was measured at 450 nm using a BioTek Synergy H4 microplate reader. A human IgG (Sigma #I2511) or IgM (Sigma #I8260-1MG) standard curve (ten three-fold serial dilutions starting at 20 µg/ml) was used to quantify samples. Wells with values >27 ng/ml were considered positive. This cutoff was determined based on our observation that the amplification of heavy and light chain variable regions always failed from cultures with a lower concentration.

ELISAs to confirm reactivity of PfMSP1_FL-3D7_-specific antibodies were performed as described above with the following modifications. Plates were coated with 50 µl in-house produced PfMSP1_FL-3D7_ per well at a concentration of 16 µg/ml (0.8 µg/well). Coated plates were incubated for 1 hour at 37°C or overnight at 4°C and all subsequent incubations were done at RT instead of 37°C. To prevent non-specific binding, the wells were blocked with 200 µl PBS containing 0.1% tween-20 and 3% non-fat milk powder (SACO), which significantly increased specificity of the assay (compared to NAP blocker). After discarding the blocking buffer from the wells, the plates were not washed. Purified antibodies were tested at a final concentration of 2.5 µg/ml in 100 – 200 µl in PBS containing 0.1% tween-20 and 1% non-fat milk powder. The plates were washed six times prior to adding the detection antibody, and four times prior to adding TMB substrate. To analyze binding strength of monoclonal antibodies to PfMSP1_FL-3D7_, chaotropic ELISAs were performed similar to the above descriptions, with the following modifications. Wells were coated with 0.2 µg protein and the final concentration of the antibodies that were tested was 0.5 µg/ml. Following the incubation with anti-MSP1 antibodies, the plates were washed four times. Then, urea (Fisher #U15-500) was added to wells at the following concentrations: 0, 1, 2, 3, 4, 5 and 8M (in 100 µl PBS with 0.1% tween-20 and 1% milk powder). After a 15-minute incubation at RT, plates were washed four times. The IC_50_ (the molar concentration of urea required to reduce antibody binding to PfMSP1 by 50%) was calculated using non-linear regression analysis in GraphPad Prism 9. Urea concentrations were log-transformed prior to analysis. OD values for each technical replicate were normalized by setting the smallest OD to 0% and the largest OD to 100%. The IC_50_ values of three technical replicates were averaged to obtain the final IC_50_ for an experiment.

ELISAs to detect antibody reactivity against merozoites were performed as described for PfMSP1-specific antibodies above with the following modifications. Free merozoites were coated at 500,000 merozoites per well in 100 µl PBS, followed by overnight incubation at 4°C. Merozoite count was estimated based on culture parasitemia. Plasma samples were tested at a 1:200 dilution in a total volume of 100 µl.

### Luminex Assay

Recombinant proteins were produced in Expi293F cells as described above, quantified using the Coomassie Plus (Bradford) Assay Kit (Thermo #23236) on a NanoDrop One spectrophotometer, according to the manufacturer’s instructions, and visualized on SDS-PAGE gel to confirm protein size and purity (**Supplementary Figures 7A, B**). 100 pmol PfMSP1_FL-3D7_, PfMSP1_FL-Dd2_, PfMSP1_FL-HB3_, PfMSP3, PfP41, Pf113, Pf3D7_1136200, PfVFT, PfAMA1, and SARS-CoV-2 receptor binding domain were coupled per 1 × 10^6^ MagPlex microspheres (Luminex, #MC10025-ID) using the Luminex protein coupling kit (#40-50016) per manufacturer’s instructions. All subsequent steps were done at RT and the beads were protected from light using aluminum foil. Coupled beads were pooled, resuspended in buffer A (PBS with 0.05% Tween 20 (Fisher #BP337), 0.5% BSA (Sigma #A7979), 0.02% sodium azide) and plated at 1000 beads per well for each protein in a black, flat-bottom 96 well plate (Bio-Rad #171025001). The beads were washed once. All washes were done with 100 µl PBST (PBS with 0.05% Tween 20) using a handheld magnetic washer (Bio-Rad #171020100). The incubation time on the magnet was always 2 min. Next, the beads were incubated with 50 µl purified anti-MSP1 antibody (diluted to 1 µg/ml using buffer B) or B cell culture supernatant (diluted 1:1 with buffer B) for 30 min. with constant agitation (500 rpm, 2.5 mm orbital diameter). Buffer B (0.05% Tween-20, 0.5% BSA, 0.02% sodium azide, 0.1% casein (Sigma #C7078), 0.5% PVA (Sigma #P8136) and 0.5% PVP (Sigma #PVP360), 15 µg/ml *E. coli* lysate) was prepared a day prior to the assay since it required the chemicals to dissolve O/N. On the day of the assay, *E. coli* lysate (MCLAB #ECCL-100) was resuspended in MQ water and added to a final concentration of 15 µg/ml. Prior to use, buffer B was centrifuged at 10,000 × g for 10 min. After three washes, 50 µl secondary antibody diluted in buffer A (PE anti-human IgG (1:200 dilution; Jackson ImmunoResearch #109-116-098)) or PE anti-human IgM (1:80 dilution; BioLegend #314507)) was added per well. After 30 min. incubation with constant agitation, the beads were washed three times and subsequently incubated in 50 µl buffer A for 30 min. with constant agitation. After one final wash, the beads were resuspended in 100 µl PBS and fluorescence intensity was measured using a calibrated and validated Bio-Rad Bio-Plex 200 machine.

### Amplification of Antibody Heavy and Light Chain Variable Regions

MSP1_FL-3D7_-specific B cells that successfully expanded in culture were collected by centrifugation (5 min. at 250 × g and RT) and stored at -70°C in 50 µl Tri-Reagent (Zymo #R2050-1-200). Heavy and light chain variable regions were amplified from PfMSP1-specific B cells by cDNA synthesis and a series of PCR reactions, shown in **Supplementary Figure 8A**. All primer sequences can be found in **Supplementary Table 4**. mRNA was isolated using Zymo’s Direct-zol RNA Microprep kit (#R2060), eluted in 15 µl elution buffer and then mixed with 0.7 µl reverse primer (10 µM, 200 mM final concentration (f/c) in 35 µl PCR reaction volume) specific for the IgG, or IgM, heavy chain (primers #7 and #297) plus 0.7 µl light chain specific reverse primers (10 µM): #108 and #109), and incubated for two minutes at 65°C. Single stranded cDNA was synthesized immediately by adding 0.7 µl SMARTScribe reverse transcriptase (100 U/µl, f/c 2 U/µl, Takara Bio #639537), 7 µl First-Strand buffer (f/c 1×), 7 µl DTT (20 mM, f/c 4 mM), 0.7 µl dNTPs (10 mM each, f/c 200 µM each, Sigma #DNTP-10), 1.75 µl RNase OUT (40 U/µl, f/c 2 U/µl, Thermo #10777019), 0.7 µl template switch oligo (TSO) (10 µM, f/c 200 nM, IDT DNA; #110, **Supplementary Table 4**), nuclease-free water till 35 µl and subsequent incubation at 42°C for 2 hours. The TSO was designed with two isodeoxynucleotides at the 5’ end to prevent TSO concatemerization and three riboguanosines at the 3’ end for increased binding affinity to the appended deoxycytidines (property of the Takara reverse transcriptase) (80, 81). The single-stranded cDNA was immediately purified using Zymo’s RNA Clean & Concentrator kit (#R1016) using Zymo’s appended protocol to purify fragments >200 nucleotides and was eluted in 10 µl elution buffer. This critical clean-up step ensured that any unused TSO was removed, preventing it from inhibiting the subsequent PCR reactions by serving as template for the forward primer. Immediately after, heavy and light chain variable regions were amplified by PCR in one reaction mix using 8.5 µl purified cDNA, 10 µl AccuStart II PCR SuperMix (QuantaBio #95137), 0.9 µl 10 µM forward primer #106 (f/c 0.45 µM, **Supplementary Table 4**), and 0.2 µl of the reverse primers (10 µM) used to synthesize the cDNA (#7, #297, #108, and #109, each at f/c 0.1 µM). Cycling conditions were 94°C for 3 min., 35 cycles of 30 sec. at 94°C, 30 sec. at 55°C and 35 sec. at 72°C, followed by 5 min. at 72°C. A second, nested amplification was required to obtain enough amplicon DNA, and was done separately for heavy chain, kappa light chain, and lambda light chain variable regions, using AccuStart II PCR SuperMix, and 2 µl of the first, unpurified PCR as template in a total reaction volume of 20 µl. Mixes of primers (**Supplementary Figure 8A**) as described by Hua-Xin Liao et al. (82) were used for this second PCR, with a final concentration of 0.1 µM for each individual primer. Reverse primer #67 was added for the heavy chain variable region PCR to allow for amplification of variable regions originating from IgG_2_, IgG_3_ and IgG_4_ mRNA, in addition to #30 which was specific for IgG_1_. Cycling conditions were as described above, except for the extension step (shortened to 30 sec.) and the annealing step, which was 30 sec. at 60°C for the IgG1 heavy chain variable region, 30 sec. at 63°C for the IgM heavy chain variable region, and 30 sec. at 50°C for the light chain variable regions.

Linear IgG expression cassettes (82) were synthesized by PCR using 3 overlapping DNA fragments: a promoter (705 bp), a variable region and a constant region (IgG_1_ heavy chain: 1188 bp; pentameric IgG_1_ heavy chain: 1326 bp; kappa light chain: 568 bp; lambda light chain: 534 bp). Details about the assembly of linear expression cassettes are described by Liao et al. (82). All fragments were amplified in a 100 µl reaction using 20 ng plasmid template [HV0023 – HV0026 (82) or the IgG_1_ expression plasmid containing the IgM multimerization sequence (see below)], 4 µl forward primer (10 µM), 4 µl reverse primer (10 µM), and QuantaBio AccuStart II PCR supermix (2×) using the following cycling program: 94°C for 3 min., 35 cycles of [94°C for 30 sec., 68°C for 30 sec., and 72°C for 40 – 75 sec. (1 min. per 1000 bp)] and 72°C for 5 min. Primers 53 and 54 were used for the promoter fragment, 55 and 58 for the heavy chain constant region fragment, 56 and 58 for the kappa light chain constant region fragment, 57 and 58 for the lambda light chain constant region fragment, and 55 and 494 for the pentameric IgG_1_ heavy chain constant region fragment (**Supplementary Table 4**). The overlapping PCRs were done as follows. The cycling program was the same for all overlapping PCRs: 98°C for 1 min., 30× (98°C for 20 sec., 68°C for 15 sec., 72°C for 60 sec.), 72°C for 10 min. Two ng of each fragment (promoter, constant region, variable region) was used as template in a 25 µl PCR reaction with 2× KAPA HiFi Hot Start Ready Mix (Roche #KK2602) and 1 µl (10 µM) of the following forward and reverse primers: 50 and 51 for the IgG1 heavy chain, 50 and 52 for the kappa and lambda light chain, and 50 and 469 for the IgG_1_ heavy chain with IgM multimerization domain (**Supplementary Table 4**). The final size of the linear expression cassettes was ~2300 bp for the IgG1 heavy chain, ~2400 bp for the IgG1 heavy chain with IgM multimerization domain, and 1600 bp for the kappa and lambda light chains (**Supplementary Figure 8B**). All linear expression cassettes were purified and sequence verified by Sanger sequencing. Variable region sequences were analyzed with IMGT/V-QUEST (38) using default settings to identify V(D)J gene usage and amino acid substitutions.

### Generation of Antibody Expression Plasmids

Antibody variable regions were cloned into expression plasmids from *In vivo*gen (#pfusess-hchg1, #pfuse2ss-hclk, #pfuse2ss-hcll2). The variable heavy and light chain regions were amplified from the linear expression cassettes (2 µl at 1 ng/µl) using 10 µl NEB Q5 Hot Start HiFi PCR master mix (#M0494S), 6 µl nuclease-free water and 1 µl sequence-specific F and R primer (10 µM, f/c 500 nM) that were based on the results of analysis using IMGT/VQUEST (38). These primers introduced restriction sites (EcoRI & NheI for hchg1, EcoRI & BsiWII for hclk, and EcoRI & AvrII for hcll2). Annealing temperatures were primer sequence dependent and were calculated using NEB’s Tm calculator to match the salt concentration in their buffer. In an attempt to express the variable regions from IgM^+^ B cells as a multimer (pentamer/hexamer mix) instead of a monomer, we modified the IgG_1_ heavy chain expression plasmid (15). The IgM multimerization sequence PTLYNVSLVMSDTAGTCY (CCAACGCTCTATAATGTCTCTTTGGTTATGTCCGACACAGCCGGTACCTGCTAT) was cloned into the IgG_1_ expression vector at the C-terminus of the open reading frame, immediately in front of the stop codon, and the leucine at position -139 relative to the proline in the multimerization sequence was changed into a cysteine. Every plasmid was Sanger sequence-verified prior to using it as expression vector.

### Antibody Expression and Purification

For small scale screening, one ml Expi293F cell cultures in a 6 well plate were transfected with heavy and light chain linear expression cassettes (1:1 molar ratio) according to the manufacturer’s instructions for 25 – 30 ml cultures (also at 125 rpm).

Heavy and light chain antibody expression plasmids were used at a molar ratio of 1:2 to transfect 5 ml cultures. The antibodies were purified from the culture supernatant 4 – 6 days later using protein G magnetic beads (Promega #G7472). Purified antibodies and antibody elution buffer [5 parts elution buffer (100 µM glycine-HCl, pH 2.7) and 1 part neutralization buffer (2M Tris buffer, pH 7.5)] were buffer exchanged to PBS using 100 kDa cutoff Protein Concentrators (Thermo #88523). The samples were diluted > 50,000 × in PBS by repeated centrifugation at 4,000 × g and 4°C. Purified antibodies were quantified using the Coomassie Plus (Bradford) Assay Kit (Thermo #23236) on a NanoDrop One spectrophotometer, according to the manufacturer’s instructions, and visualized on SDS-PAGE gel with a standard amount of BSA to confirm protein size and purity (**Supplementary Figure 7C**).

### Immunofluorescence Assay

All steps of the immunofluorescence assay were done at RT. A thin blood smear was made on a microscopy slide from a 1 µl drop of E64-treated schizont culture. After drying for 30 sec., the cells were fixed by loading 1 ml of 4% paraformaldehyde (Electron Microscopy #15710) on the slide and incubating it for 30 min. The fixed cells were then washed three times with 1 ml PBS. Following the washes, the cells were permeabilized with 0.1% Triton-X (Fisher # BP151) in PBS and incubated for 30 min. The cells were then washed for an additional three washes using PBS. The slide was treated with blocking buffer (2% BSA, 0.05% Tween-20, 100 mM Glycine, 3 mM EDTA and 150 mM NaCl in PBS) for one hour. PfMSP1-specific mAbs were added to the cells at 1 µg/ml in 500 µl blocking buffer and incubated for one hour. Samples were then washed again three times using PBS. Goat anti-human IgG conjugated to FITC (Thermo #A18830) secondary antibody was diluted 1:1000 (1.5 µg/ml) in blocking buffer and then added to the smear to incubate for one hour in the dark. Samples were again washed three times using PBS in the dark and then allowed to air dry for one hour in the dark. Slides were mounted using 10 µl ProLong Glass mounting medium containing NucBlue Stain (Thermo #P36985) and sealed with a cover slip. Samples were imaged using a Zeiss Axio Imager Z1 with Zen Blue software.

### Growth Inhibition Assay

*Pf* isolate 3D7 parasites were pre-synchronized at the ring stage with a 5% D-sorbitol (Fisher #S459-500) treatment as described above, followed four days later by two additional 5% D-sorbitol treatments 14 hours apart (76). At the late trophozoite/early schizont stage (24 hours after the third D-sorbitol treatment), parasitemia was determined by inspection of a Giemsa-stained blood smear. The smear was also used to confirm correct parasite staging. Immediately after, 20 µl of each antibody (1 mg/ml in PBS) was added to wells containing 30 µl complete medium in a black clear bottom 96-well plate (Corning #3603). A monoclonal antibody specific for apical membrane antigen 1 (AMA1) was used as a positive control (BEI #MRA-481A). Antibody elution buffer (100 mM glycine-HCl, pH 2.7) that was buffer exchanged to PBS alongside purified antibodies (see “Antibody expression and purification” above) was used as a negative control. Fifty µl parasite culture (1% parasitemia and 2% hematocrit) was then added to wells containing antibody or negative control. Uninfected erythrocytes (2% hematocrit) were used to determine the background signal. The plate was then incubated at standard parasite culture conditions (described above) for 48 hours before being transferred to a -70°C freezer. After overnight incubation of the plate at -70°C, SYBR green dye (Invitrogen #S7585) was added to lysis buffer (20 mM Tris-HCl (pH7.5), 5 mM EDTA, 0.008% saponin (Sigma # 558255100GM), 0.08% Triton X-100 in MQ water) at 0.2 µl dye per ml of lysis buffer. One hundred µl SYBR green lysis buffer was added to each well and the plate was incubated in the dark at 37°C for 3 – 6 hours. Fluorescence (excitation = 495 nm, emission = 525 nm, cutoff = 515 nm) was measured with a BioTek Synergy H4 plate reader. The instrument was programmed to read the plate from the bottom after mixing for 5 sec. The average background fluorescence value was subtracted from the fluorescence signal of the wells with infected cells. Percent growth inhibition was expressed as the reduction in fluorescence signal in wells incubated with antibody as compared to the negative control.

### Plasma Antibody Proteomics

Total IgG was isolated from 1 ml plasma using Protein G Plus Agarose (Thermo #22851) affinity chromatography and cleaved into F(ab’)_2_ fragments using IdeS protease. PfMSP1-specific F(ab’)_2_ was isolated by affinity chromatography using 1 mg recombinant PfMSP1_19-3D7_ produced in *E. coli* (Meridian Life Sciences, #R01603 and #R01604) coupled to 0.05 mg dry NHS-activated agarose resin (Thermo #26196). This is a large excess of antigen, i.e., a nearly 1:1 molar ratio of antigen to total IgG, of which only a fraction will bind to PfMSP1_19-3D7_. F(ab’)_2_ (10 mg/ml in PBS) was rotated at 8 rpm with antigen-conjugated affinity resin for 1 hour at RT, loaded into 0.5 ml spin columns (Thermo #89868), washed 12× with 0.4 ml Dulbecco’s PBS (1,000 × *g* for 30 sec. at RT), and eluted with 0.5 ml fractions of 1% formic acid. IgG-containing elution fractions were concentrated to dryness in a speed-vac, resuspended in ddH_2_O, combined, neutralized with 1M Tris/3M NaOH. Success of the affinity purification was assessed by confirming the depletion of anti-MSP1_19-3D7_ F(ab’)_2_ in the flow-through. The sample was then prepared for liquid chromatography–tandem mass spectrometry (LC-MS/MS) as described previously (83, 84) with the modifications that (i) peptide separation using acetonitrile gradient was run for 120 min and (ii) data was collected on an Orbitrap Fusion (Thermo Fisher Scientific) operated at 120,000 resolution using HCD (higher-energy collisional dissociation) in topspeed mode with a 3 sec. cycle time. B cell receptor sequencing data was available from a previous study (43). Demultiplexing of sequence reads and the generation of consensus sequences for UMI groups were performed as outlined by Turchaninova et al. using software tools MIGEC (v1.2.9) and MiTools (v1.5) (85). Sequences with ≥ 2 reads were clustered into clonal lineages defined by 90% HCDR3 amino acid identity using USEARCH (86). LC-MS/MS search databases were prepared as previously described (83), using custom Python scripts (available upon request). MS searches, and MS data analyses were performed as previously described (83, 84), adjusting the stringency of the elution XIC:flowthrough XIC filter to 2:1.

### Data Visualization and Statistics

Flow cytometry data were analyzed and plotted using FlowJo (v10.7.1). Dot plots were generated using the package ggplot2 in RStudio (v1.4.1103) using R (v4.0.4). All other plots were generated in GraphPad Prism 9, which was also used for statistical analyses. The statistical test used for each analysis is indicated in the figure legends.

## Data Availability Statement

The datasets presented in this study can be found in online repositories. The names of the repository/repositories and accession number(s) can be found below: MassIVE, MSV000088532.

## Ethics Statement

The studies involving human participants were reviewed and approved by the Makerere University School of Medicine Research and Ethics Committee (SOMREC), the University of California, San Francisco Human Research Protection Program & IRB, and the Institutional Review Board of the University of Texas Health Science Center at San Antonio. Written informed consent to participate in this study was provided by the participants’ legal guardian/next of kin.

## Author Contributions

EB secured funding for the study, conceived the research question, and designed the study. SG performed flow cytometry. SG, KC, and SB produced recombinant antigens and monoclonal antibodies and contributed to other experiments. GB performed IFA and growth-inhibition assays. SG and RG performed Luminex experiments. RR, RG, and AB performed merozoite ELISAs. KG and GI performed plasma IgG proteomics. IS and BG provided clinical samples and data. SG, SB, and EB wrote the manuscript with input from all other co-authors. All authors contributed to the article and approved the submitted version.

## Funding

This work was supported by National Institutes of Health/National Institute of Allergy and Infectious Diseases (R01 AI153425 to EB). SG and AB were supported by Graduate Research in Immunology Program training grant NIH T32 AI138944. RR was supported by Translational Science Training award TL1 TR002647. Data were generated in the Flow Cytometry Shared Resource Facility, which is supported by UT Health, NIH-NCI P30 CA054174-20 (CTRC at UT Health) and UL1 TR001120 (CTSA grant) and in the Genome Sequencing Facility, which is supported by UT Health San Antonio, NIH-NCI P30 CA054174 (Cancer Center at UT Health San Antonio), NIH Shared Instrument grant 1S10OD021805-01 (S10 grant), and CPRIT Core Facility Award (RP160732).

## Conflict of Interest

The authors declare that the research was conducted in the absence of any commercial or financial relationships that could be construed as a potential conflict of interest.

## Publisher’s Note

All claims expressed in this article are solely those of the authors and do not necessarily represent those of their affiliated organizations, or those of the publisher, the editors and the reviewers. Any product that may be evaluated in this article, or claim that may be made by its manufacturer, is not guaranteed or endorsed by the publisher.

## Abbreviations

BCR, B cell receptor; HCDR3, heavy chain complementarity determining region 3; Ig, immunoglobulin; MBC, memory B cell; mAb, monoclonal antibody.
