# Supplementary material for: A Molecular Analysis of Memory B Cell and Antibody Responses Against Plasmodium falciparum Merozoite Surface Protein 1 in Children and Adults From Uganda
Source: Front Immunol. 2022 Jun 2;13:809264. doi: 10.3389/fimmu.2022.809264 (PMC9201334; doi:10.3389/fimmu.2022.809264)
Supplement: Supplementary file 1 [file DataSheet_1.pdf]

A molecular analysis of memory B cell and antibody responses against *Plasmodium falciparum* merozoite surface protein 1 in children and adults from Uganda

S. Jake Gonzales[1], Kathleen N. Clarke[1], Gayani Batugedara[1], Rolando Garza[1], Ashley E. Braddom[1], Raphael A. Reyes[1], Isaac Ssewanyana[2,3], Kendra C. Garrison[4], Gregory C. Ippolito[5], Bryan Greenhouse[6], Sebastiaan Bol[1], and Evelien M. Bunnik[1]*

[1] Department of Microbiology, Immunology and Molecular Genetics, Long School of Medicine, The University of Texas Health Science Center at San Antonio, San Antonio, TX, USA
[2] Infectious Disease Research Collaboration, Kampala, Uganda
[3] London School of Hygiene and Tropical Medicine, London, UK
[4] Department of Chemical Engineering, University of Texas at Austin, Austin, TX, USA
[5] Department of Molecular Biosciences and Department of Oncology, Dell Medical School, University of Texas at Austin, Austin, TX 78712, USA
[6] Department of Medicine, University of California San Francisco, San Francisco, CA, USA

***Correspondence**
Evelien M. Bunnik, Ph.D.
bunnik@uthscsa.edu
7703 Floyd Curl Drive
San Antonio, TX 78229
United States of America

Supplementary table 1: Characteristics of donors included in the study

Supplied as a supplementary Excel file.

Supplementary table 2: Molecular characteristics of anti-MSP1 plasma IgG as determined by proteomics analysis

Abun-dance (%)	Size of B cell lineage[1]	B cell subset[2]	Isotype	V-gene (IGH)	J-gene (IGH)	Amino acid changes in V-gene (%)	HCDR3 length (amino acids)
24.7	1	cMBC	IGHG3	V3-53	J5	22	10
23.5	5	cMBC	IGHG3	V1-69	J6	26	24
15.5	1	cMBC	IGHG3	V3-64	J1	25	18
14.0	1	cMBC	IGHA1	V4-28	J5	16	10
6.9	4	cMBC	IGHG1	V1-69	J6	25	21
4.5	1	NBC	IGHD	V1-3	J5	0	15
3.1	1	atMBC	IGHG3	V1-24	J4	3	19
2.9	1	cMBC	IGHG3	V3-23	J4	19	20
2.5	1	NBC	IGHM	V4-28	J5	7	18
1.2	1	cMBC	IGHG1	V4-39	J4	22	15
0.4	1	NBC	IGHM	V4-59	J3	0	8
0.4	1	cMBC	IGHG3	V1-3	J3	25	16
0.2	1	cMBC	IGHG3	V4-34	J5	20	18
0.1	1	atMBC	IGHM	V3-33	J4	2	11
<0.1	2	atMBC	IGHM	V3-30	J4	13	20
<0.1	1	NBC	IGHM	V3-53	J3	0	9
<0.1	1	NBC	IGHM	V3-23	J5	0	16
<0.1	1	NBC	IGHD	V3-53	J4	0	14

[1] Number of unique clonal sequences found in BCR-seq data set that belonged to the same clonal lineage as the plasma IgG detected by proteomics analysis.

[2] BCR-seq subset in which a match was found with the sequence of anti-MSP1 plasma IgG.

Note: the lineage to which mAb10 and mAb22 belong is highlighted in green.

cMBC, classical memory B cell; NBC, naïve B cell; atMBC, atypical MBC.

Supplementary table 3: Antibodies used for flow cytometry

Antibody	Fluorophore	Clone	Company / catalog number
CD19	BV421	SJ25C1	BioLegend / 363017
CD20	BV785	2H7	BioLegend / 302355
CD21	PerCP-eF710	HB5	Thermo / 46021942
CD27	PE-Cy7	O323	Thermo / 25027941
IgA	FITC	IS11-8E10	Miltenyi / 130-099-107
IgD	PE-Dazzle594	IA6-2	BioLegend / 348240
IgG	FITC	G18-145	BD / 560952
IgM	BV711	MHM-88	BioLegend / 314540
IgM	eFluor 450	SA-DA4	Thermo / 48999841

Supplementary table 4: Primers for amplification of antibody variable regions and the construction of linear expression cassettes

#	Sequence (5'-3')[1]
7	CGCCTGAGTTCCACGACACC
24	**CTGGGTTCCAGGTTCCACTGGTGAC**CAGGTGCAGCTGGTRCAGTCTGGG
25	**CTGGGTTCCAGGTTCCACTGGTGAC**CAGRGCACCTTGARGGAGTCTGGTCC
26	**CTGGGTTCCAGGTTCCACTGGTGAC**GAGGTKCAGCTGGTGGAGTCTGGG
27	**CTGGGTTCCAGGTTCCACTGGTGAC**CAGGTGCAGCTGCAGGAGTCGG
28	**CTGGGTTCCAGGTTCCACTGGTGAC**GARGTGCAGCTGGTGCAGTCTGGAG
29	**CTGGGTTCCAGGTTCCACTGGTGAC**CAGGTACAGCTGCAGCAGTCAGGTCC
30	GCTGTGCCCCCAGAGGTGCTCYTGGA
31	**CTGGGTTCCAGGTTCCACTGGTGAC**GACATCCAGWTGACCCAGTCTC
32	**CTGGGTTCCAGGTTCCACTGGTGAC**GATATTGTGATGACCCAGWCTCCAC
33	**CTGGGTTCCAGGTTCCACTGGTGAC**GAAATTGTGTTGACRCAGTCTCCA
34	**CTGGGTTCCAGGTTCCACTGGTGAC**GACATCGTGATGACCCAGTCTC
35	**CTGGGTTCCAGGTTCCACTGGTGAC**GAAACGACACTCACGCAGTCTC
36	**CTGGGTTCCAGGTTCCACTGGTGAC**GAAATTGTGCTGACWCAGTCTCCA
37	**CTGGGTTCCAGGTTCCACTGGTGAC**GACATTGTGCTGACCCAGTCT
38	GGGAAGATGAAGACAGATGGT
39	**CTGGGTTCCAGGTTCCACTGGTGAC**CAGTCTGTGYTGACKCAGCC
40	**CTGGGTTCCAGGTTCCACTGGTGAC**CAGTCTGCCCTGACTCAGCC
41	**CTGGGTTCCAGGTTCCACTGGTGAC**TCYTATGAGCTGACWCAGCCAC
42	**CTGGGTTCCAGGTTCCACTGGTGAC**TCTTCTGAGCTGACTCAGGACCC
43	**CTGGGTTCCAGGTTCCACTGGTGAC**CAGCYTGTGCTGACTCAATC
44	**CTGGGTTCCAGGTTCCACTGGTGAC**CTGCCTGTGCTGACTCAGC
45	**CTGGGTTCCAGGTTCCACTGGTGAC**CAGSCTGTGCTGACTCAGCC
46	**CTGGGTTCCAGGTTCCACTGGTGAC**AATTTTATGCTGACTCAGCCCCACT
47	**CTGGGTTCCAGGTTCCACTGGTGAC**CAGRCTGTGGTGACYCAGGAG
48	**CTGGGTTCCAGGTTCCACTGGTGAC**CAGGCAGGGCWGACTCAG
49	GGGYGGGAACAGAGTGACC
50	AGTAATCAATTACGGGGTCATTAGTTCATAG
51	TCCCCAGCATGCCTGCTATTGTCTTCCCAATC
52	TCCCCAGCATGCCTGCTATTGTC

53	ATCCACTAGTAACGGCCGCCAGTG
54	CTGGGTTCCAGGTTCCACTGGTGAC
55	CACCTCTGGGGGCACAGC
56	CGAACTGTGGCTGCACCATCTGTCTTCATC
57	TGCCCCCTCGGTCACTCTGTTCCCGCCC
58	CAGTCACGACGTTGTAAAACGACG
67	GCTGTGCTCTCGGAGGTGCTCCTGGA
106	CAGCAGTGAGTAGAACCGTATCCG
108	ATGGCGGGAAGATGAAGACAG
109	AGTGTGGCCTTGTTGGCTTG
110	5-Me-isodC/iso-dGCAGCAGTGAGTAGAACCGTATCCGrGrGrG
297	CCGACGGGGAATTCTCACAG
298	GCTGTGCCCCCAGAGGTGGAATTCTCACAGGAGACGAGG
469	GTAGAGGCTTGATTTGGAGGT
494	TGCCTATGCCTTATTCATCCCTC

[1] Sequences indicated in bold do not bind to template but provide complementarity to the promoter or constant regions during the overlapping PCR to generate linear expression cassettes.

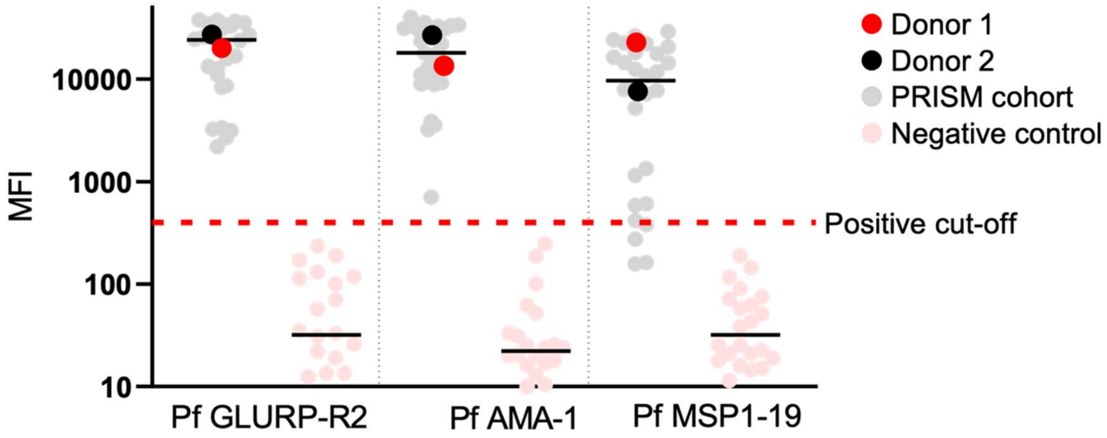

Supplementary figure 1: Plasma antibody reactivity against *P. falciparum* antigens in malaria-experienced blood donors. Antibody titers against three different parasite antigens were assessed by Luminex assay on plasma from malaria-experienced Ugandan blood donors (red and black). As a reference, antibody reactivity among adult participants (≥18 years of age) of the PRISM cohort who live in a region of high *P. falciparum* transmission are also provided (n = 28; gray). None of the participants had symptomatic malaria at the selected routine visit time point, but all participants had well-documented prior asymptomatic and symptomatic *P. falciparum* infections. Negative control samples were obtained from European adults (≥18 years of age) who had never traveled to malaria-endemic countries (n = 22; pink). Black lines represent the median MFI of the PRISM cohort samples and the negative control samples. The mean of the negative control samples plus two standard deviations was used as the positive cutoff value. For all three antigens, the cutoff value was similar. The GLURP-R2 cutoff was the highest, and this value was chosen as the positive cutoff for the graph (dashed red line).

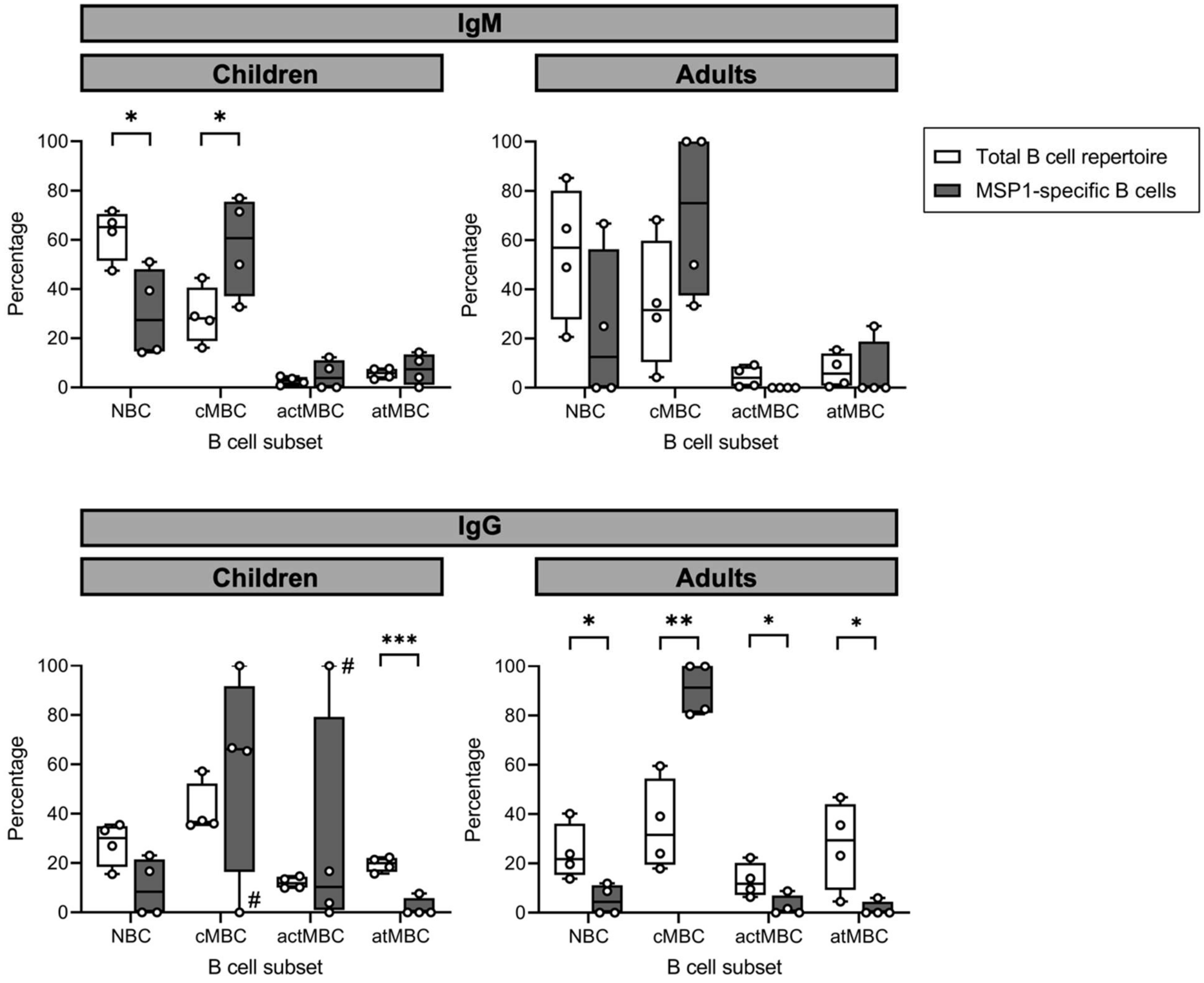

Supplementary figure 2: Relative abundance of major B cell subsets among the total B cell repertoire and among MSP1-specific B cells in partially immune children (n = 4) and immune adults (n = 4). The four major B cell subsets are defined as follows: naïve B cells (NBC), $CD21^+$ $CD27^-$; classical memory B cells (cMBC), $CD21^+$ $CD27^+$; activated memory B cells (actMBC), $CD21^-$ $CD27^+$; and atypical memory B cells (atMBC), $CD21^-$ $CD27^-$. Data are split out by isotype, showing IgM^+ B cells in the top and IgG^+ B cells in the bottom. The data points labeled with # in the bottom left plot represent a small number of IgG^+ B cells in a child that had a malaria episode four weeks prior to sample collection. All IgG^+ B cells detected in this individual had an activated memory B cell phenotype. *** $P < 0.001$; ** $P < 0.01$; * $P < 0.05$

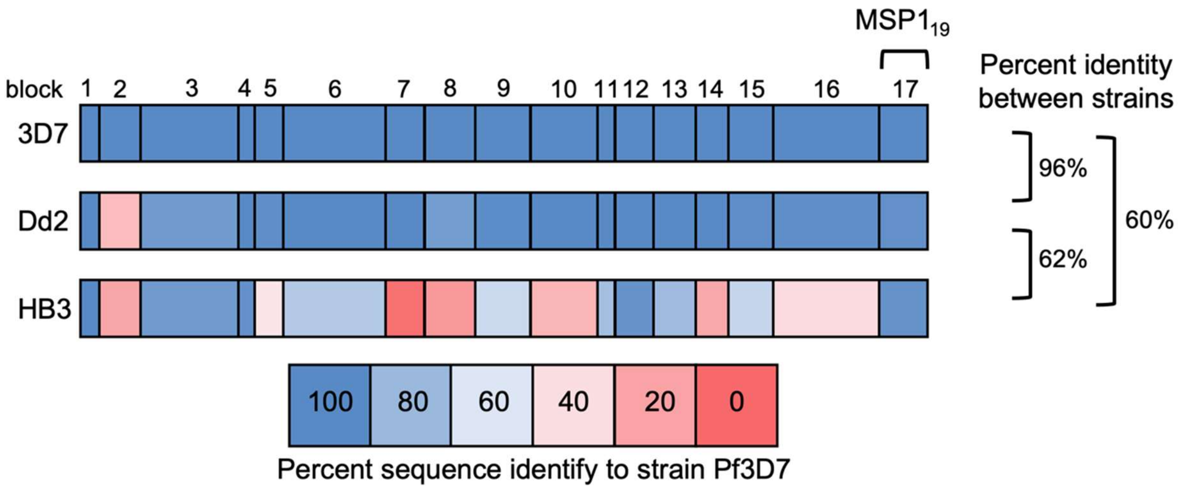

Supplementary figure 3: Schematic overview of the sequence identity of MSP1 between *P. falciparum* strains 3D7, Dd2, and HB3. Protein sequences were obtained from PlasmoDB, separated into blocks based on the description by Tanabe *et al.* (J. Mol. Biol., 1987), and aligned using the EMBL-EBI tool EMBOSS Needle with default settings (Needleman and Wunsch, J. Mol. Biol, 1970). The percent sequence identity calculated by this tool was used to color-code the block regions of MSP1.

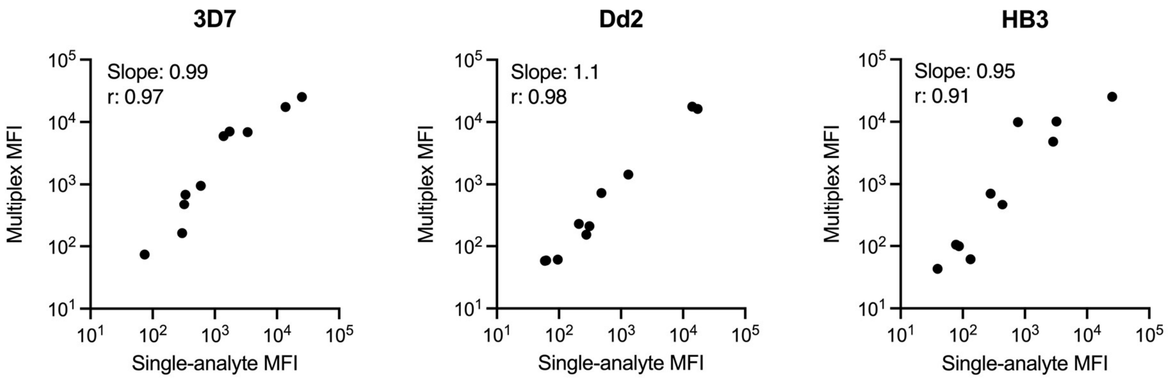

Supplementary figure 4: MSP1 variant multiplex assay validation. Malaria-experienced donor-derived monoclonal MSP1 antibodies (n = 10) were tested for reactivity against MSP1 variants (3D7, Dd2, and HB3) via multiplex and single-analyte Luminex assay. Result shown are mean fluorescence intensity (MFI) from MSP1 variant single-analyte assay (Single-analyte MFI) and corresponding MFI from MSP1 variant multiplex assay (Multiplex MFI). Statistics shown are slope by simple linear regression and Pearson r.

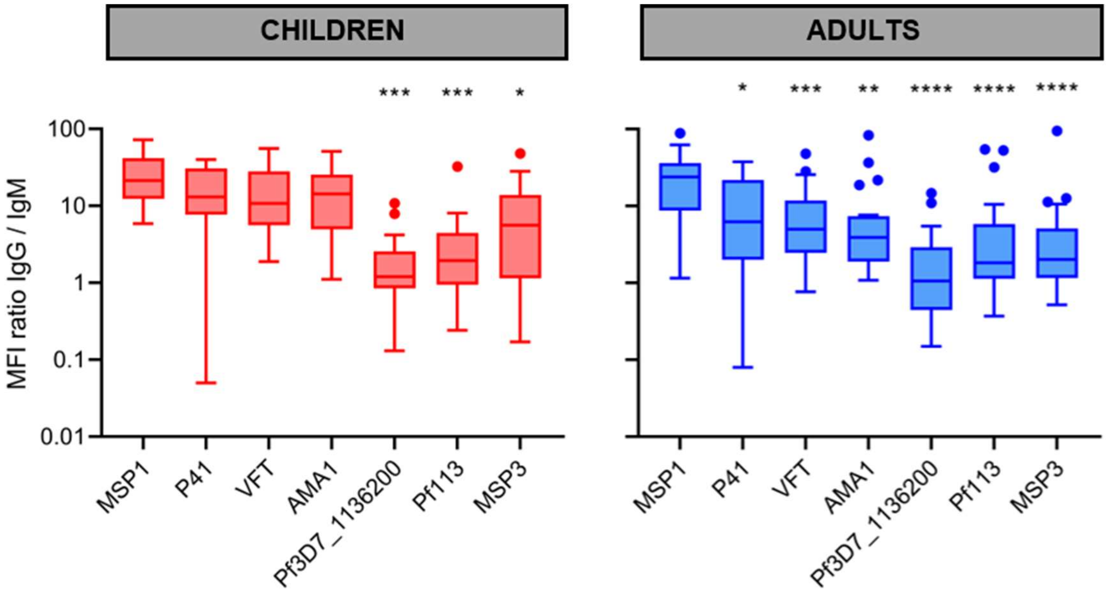

Supplementary figure 5: Comparison of plasma IgG versus IgM reactivity between MSP1$_{FL-3D7}$ and a panel of six other merozoite antigens. Data points shown are the ratio of plasma IgG and IgM Luminex MFI values for malaria-experienced children (n = 18, left) and adults (n = 24, right). The IgG/IgM ratios of MSP1$_{FL-3D7}$ were compared with all other antigens using a repeated measures one-way ANOVA with Geisser-Greenhouse correction, followed by a Dunnett's multiple comparisons test. **** $P < 0.0001$; *** $P < 0.001$; ** $P < 0.01$; * $P < 0.05$

Heavy chains

```
IGHV1-69*06 + IGHJ6*02
         <         FR1           >< CDR1 ><      FR2      >< CDR2 >
Germline QVQLVQSGAEVKKPGSSVKVSCKASGGTFSSYAISWVRQAPGQGLEWMGGIIPIFGTA
mAb10    ..............................N.P....................V.A.T
mAb22    ............R..........I..DS.FNFP.....R..............V.A.T

         <              FR3                   ><         CDR3      ><   FR4   >
Germline NYAQKFQGRVTITADKSTSTAYMELSSLRSEDTAVYYCAR           YYYYGMDVWGQGTTVTVSS
mAb10    .......D................V...K.............ALSRVRGVITH.............A.....
mAb22    D.SLR..D..A....Q..N......TN.KP..S.F.F.V.AVTRVRGVIVHH...AL....P........
```

Light chains

```
IGKV3-11*01 + IGKJ4*01
         <          FR1           ><CDR1><      FR2      ><2>
Germline EIVLTQSPATLSLSPGERATLSCRASQSVSSYLAWYQQKPGQAPRLLIYDAS
mAb10    .............................DN........A......I.....

         <              FR3                 >< CDR3 ><  FR4   >
Germline NRATGIPARFSGSGSGTDFTLTISSLEPEDFAVYYCQQRSN LTFGGGTKVEIK
mAb10    K...........G...................L.......DE......N....R
```

```
IGLV2-14*03 + IGJL2*01
         <         FR1           ><  CDR1 ><      FR2      ><2>
Germline QSALTQPASVSGSPGQSITISCTGTSSDVGGYNYVSWYQQHPGKAPKLMIYEVS
mAb22    ........................STG.I.Y..............VIL.Q.N

         <              FR3                 >< CDR3  ><  FR4   >
Germline NRPSGVSNRFSGSKSGNTASLTISGLQAEDEADYYCSSYTSS VLFGGGTKLTVL
mAb22    .......D...........Y.......DD.D...F.C.H...M............
```

Supplementary figure 6: Heavy and light chain variable region sequences of mAb10 and mAb22. The amino acid sequences of both monoclonal antibodies are aligned to their respective germline V-gene and J-gene sequences. Framework regions are indicated in blue and CDRs are indicated in yellow. Amino acid changes relative to the heavy chain variable region germline sequence that are shared between mAb10 and mAb22 are highlighted in orange.

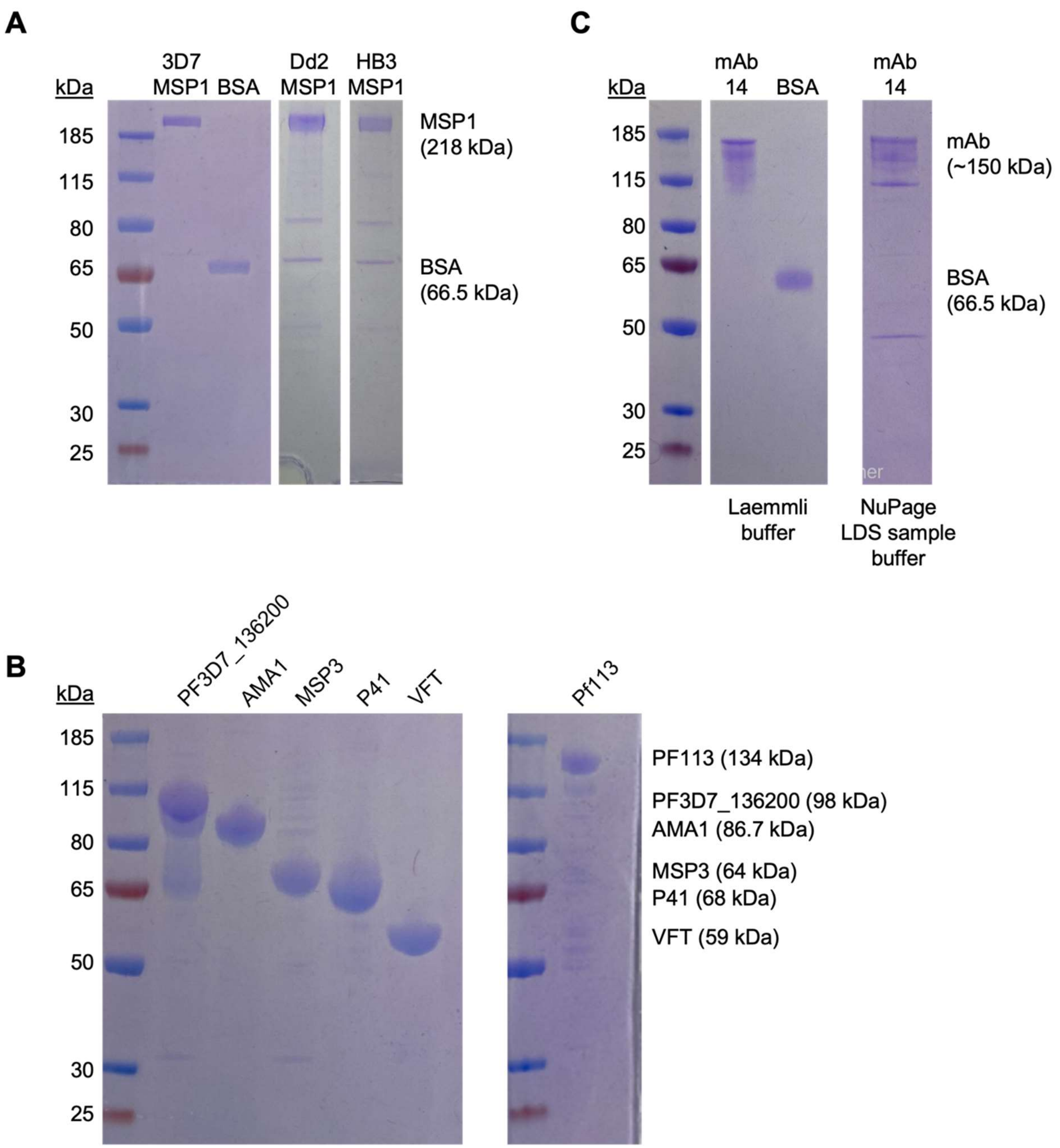

Supplementary figure 7: Quality control of recombinant MSP1 and monoclonal antibodies by SDS-PAGE. A) MSP1$_{FL-3D7}$, BSA, MSP1$_{FL-Dd2}$ and MSP1$_{FL-HB3}$ (0.8 – 1.5 µg). **B)** *P. falciparum* PF3D7_1136200, AMA1, MSP3, P41, VFT (all 3 µg), and Pf113 (2 µg). **C)** mAb14 and BSA (0.5 - 1 µg). All proteins were loaded on gel in Laemmli buffer, with NuPage sample reducing agent for MSP1, but not mAbs. Loading mAbs in NuPage LDS sample buffer resulted in a pattern that resembled impartially formed immunoglobulins (mAb14 shown on the left in panel B).

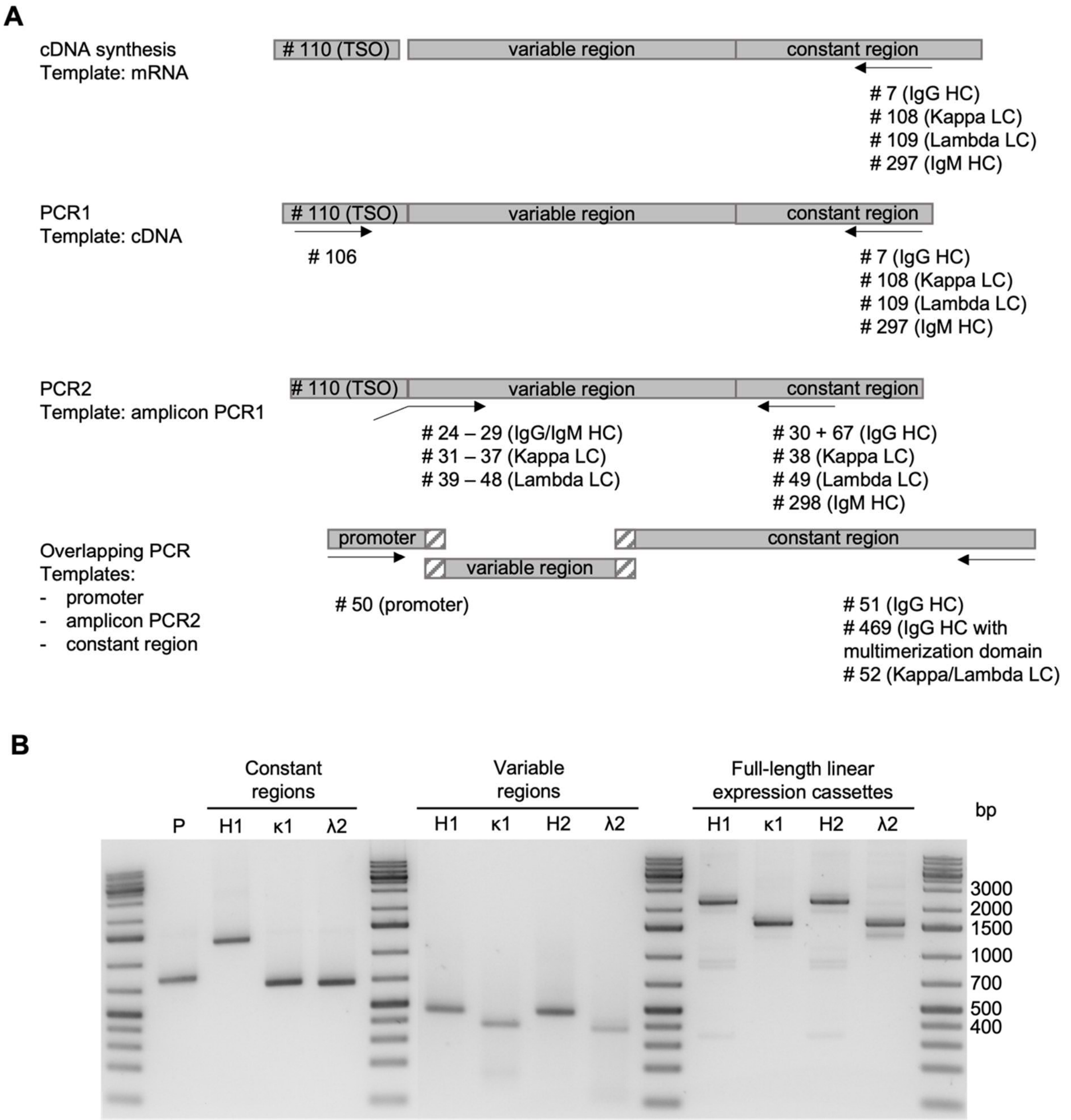

Supplementary figure 8: Generation of linear antibody expression cassettes. A) Schematic representation of reverse transcription and amplification of heavy and light chain variable regions from B cell mRNA. The sequences of primers indicated are listed in **Supplementary table 5**. **B)** DNA electrophoresis images showing the individual amplicons used in the overlapping PCR (P, promoter; H, heavy chain; κ, kappa light chain; λ, lambda light chain). The last four lanes show the full-length linear expression cassettes used for the expression of two recombinant monoclonal antibodies, one with a kappa light chain (H1 and κ1) and one with a lambda light chain (H2 and λ2).
